# Use of machine learning and Poincaré density grid in the diagnosis of sinus node dysfunction caused by sinoatrial conduction block in dogs

**DOI:** 10.1111/jvim.17071

**Published:** 2024-04-29

**Authors:** Wyatt Hutson Flanders, N. Sydney Moïse, Niels F. Otani

**Affiliations:** ^1^ Department of Clinical Sciences, College of Veterinary Medicine Cornell University Ithaca New York USA; ^2^ Section of Cardiology, Department of Clinical Sciences, College of Veterinary Medicine Cornell University Ithaca New York USA; ^3^ School of Mathematical Sciences Rochester Institute of Technology Rochester New York USA

**Keywords:** 24‐hour electrocardiography, artificial intelligence, bradycardia, exit block, Holter monitoring, parasympathetic, sinoatrial conduction pathways, sinus pauses

## Abstract

**Background:**

Sinus node dysfunction because of abnormal impulse generation or sinoatrial conduction block causes bradycardia that can be difficult to differentiate from high parasympathetic/low sympathetic modulation (HP/LSM).

**Hypothesis:**

Beat‐to‐beat relationships of sinus node dysfunction are quantifiably distinguishable by Poincaré plots, machine learning, and 3‐dimensional density grid analysis. Moreover, computer modeling establishes sinoatrial conduction block as a mechanism.

**Animals:**

Three groups of dogs were studied with a diagnosis of: (1) balanced autonomic modulation (n = 26), (2) HP/LSM (n = 26), and (3) sinus node dysfunction (n = 21).

**Methods:**

Heart rate parameters and Poincaré plot data were determined [median (25%‐75%)]. Recordings were randomly assigned to training or testing. Supervised machine learning of the training data was evaluated with the testing data. The computer model included impulse rate, exit block probability, and HP/LSM.

**Results:**

Confusion matrices illustrated the effectiveness in diagnosing by both machine learning and Poincaré density grid. Sinus pauses >2 s differentiated (*P* < .0001) HP/LSM (2340; 583‐3947 s) from sinus node dysfunction (8503; 7078‐10 050 s), but average heart rate did not. The shortest linear intervals were longer with sinus node dysfunction (315; 278‐323 ms) vs HP/LSM (260; 251‐292 ms; *P* = .008), but the longest linear intervals were shorter with sinus node dysfunction (620; 565‐698 ms) vs HP/LSM (843; 799‐888 ms; *P* < .0001).

**Conclusions:**

Number and duration of pauses, not heart rate, differentiated sinus node dysfunction from HP/LSM. Machine learning and Poincaré density grid can accurately identify sinus node dysfunction. Computer modeling supports sinoatrial conduction block as a mechanism of sinus node dysfunction.

**Abbreviations**
bpm: beats per minute
cRMSSD: corrected root mean square of successive differences
ECG: electrocardiogram
HP/LSM: high parasympathetic/low sympathetic modulation
ms: milliseconds
s: seconds

## 1 INTRODUCTION

**FIGURE 1:**
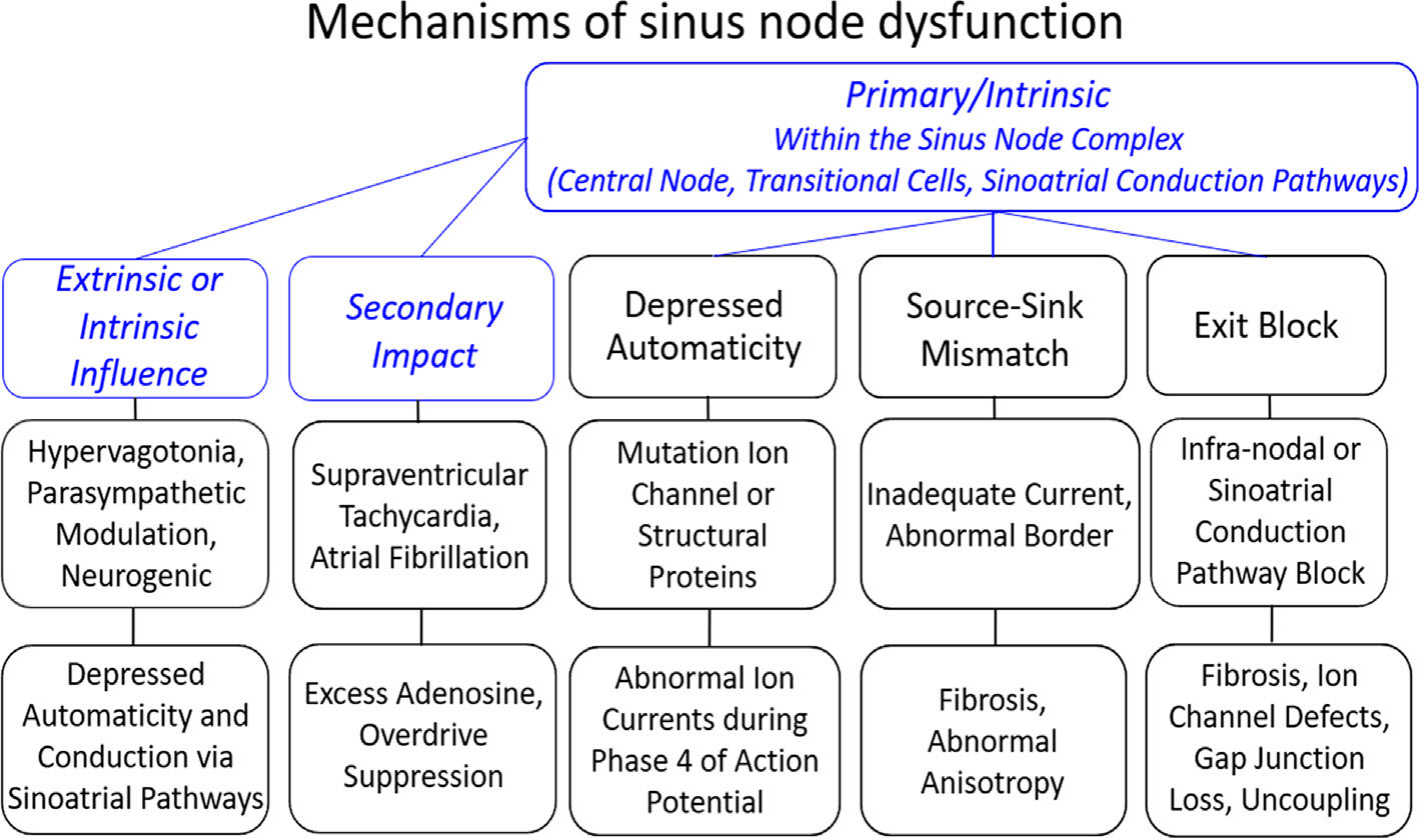
Potential mechanisms of sinus node dysfunction. Diseases of the sinus node may involve a primary defect of decreased spontaneous depolarization (depressed automaticity), inability to excite the atrial myocardium (source‐sink mismatch) or conduction block within the sinus node or the sinoatrial conduction pathways (exit block). Furthermore, more than 1 reason can be present. Compromised pacing or conducting cells may be impacted to a greater degree by extrinsic influences or the consequences of atrial tachycardias. Genetics, aging and drugs can impact sinus node function and conduction in the sinoatrial conduction pathways. This study selected for dogs with sinus node dysfunction that had beat‐to‐beat evidence consistent with a component of exit block from the sinus node.

The pacing cells of the sinus node represent <0.0004% of the billions of myocytes in the heart^1^; and yet, the architecture and molecular composition of this small structure ensure life with a stoichiometric redundancy of function that initiates the depolarization of the entire heart.^2^ Two reasons permit the few pacing cells to depolarize the large atrial mass. The pacing cells synchronize to generate an impulse of sufficient strength to propagate through sinoatrial conduction pathways to excite the atrial myocardium.^3^, ^4^ Additionally, the sinus node and conduction pathways contain fibrous tissue^5^ and connexin 45^2^, ^6^ and no connexin 43^7^ resulting in low conductance. The slowed conduction from the sinus node to the atria permits charge to build and overcome the source‐sink difficulty.^2^, ^8^ However, these features that assure enough current to overcome the source‐sink challenge are also those that are vulnerable to alterations that slow impulse propagation leading to sinus node dysfunction (Figure 1).^9^, ^10^, ^11^, ^12^

Aging and disease insult the sinus node with decreased pacing cells, increased fibrosis, excessive adipose infiltrate, plus altered components of protein expression and ion channels.^2^, ^6^, ^9^, ^10^, ^13^, ^14^, ^15^, ^16^, ^17^, ^18^ This destruction from the molecular to the cellular levels leads to electrocardiographically recognized manifestations of sinus node dysfunction. These include sinus bradycardia, sinus pauses, sinus arrest, sinoatrial conduction pathway block (exit block),^3^, ^11^, ^12^, ^17^, ^19^, ^20^ sinus node reentrant tachycardia, and atrial tachycardia.^2^, ^3^, ^4^, ^6^, ^18^, ^21^ Clinically relevant, the damaged sinus node and pathways are more vulnerable to autonomic modulation.^22^

Distinguishing sinus node dysfunction from high parasympathetic/low sympathetic modulation (HP/LSM) can be challenging in the dog.^20^, ^23^, ^24^, ^25^, ^26^ Newer methods of analysis that plot the RR intervals against the next RR interval (Poincaré plots)^27^, ^28^, ^29^ can provide not only evidence for the diagnosis, but indicators of the mechanisms for sinus node dysfunction.^25^, ^30^, ^31^ If beat‐to‐beat patterns are visually identified on Poincaré plots, then objective determinations should be possible. Nevertheless, until the advent of machine learning,^32^, ^33^, ^34^, ^35^, ^36^, ^37^, ^38^, ^39^ rule‐based quantification of Poincaré plots has been difficult^40^, ^41^ and imperfect for use in the dog.^23^, ^25^ Machine learning without specific algorithms of computer programming and creation of a 3‐dimensional beat‐to‐beat grid for matching intervals may better enable rhythm identification.

We hypothesized that specific heart rate parameters, beat‐to‐beat patterns, and computer algorithms could identify sinus node dysfunction with sinoatrial conduction block in the dog. Beat intervals taken from 24‐hour ECGs were studied from dogs with: (1) heart rates between 72 and 96 bpm, (2) HP/LSM reflected by heart rates <65 bpm and (3) hypothesized exit block causing sinus node dysfunction. The objectives were to: (1) identify differentiating heart rate parameters, (2) quantify features of Poincaré plots, (3) train and test machine learning and Poincaré density grid algorithms to diagnose the rhythms, and (4) create a computer model that supported sinoatrial conduction block as a mechanism for sinus node dysfunction.

## 2 MATERIALS AND METHODS

### 2.1 Selection of 24‐hour ECG data

Holter recordings were reviewed from 6 veterinary hospitals that permitted export of data to the Trillium Holter Analysis Software (Forest Medical, Syracuse, New York).

Recording criteria included: (1) quality 3‐lead ECG with <1% artifact, (2) <5 P waves without a QRS complex (very low grade 2nd degree atrioventricular nodal conduction block), (3) a consistent PQ/PR interval permitting the RR interval to serve as a surrogate for the PP interval (gradual increase of <60 ms in the PQ/PR interval during sleep permitted), (4) >22 hours recorded, (5) <10% of QRS complexes were escape beats, and (6) <200/24‐hours premature complexes.

The goal was to obtain 24‐hour recordings with 1 of 3 diagnoses: (1) balanced autonomic modulation [average 24‐hour heart rate between 74 and 96 beats per minute (bpm)], (2) HP/LSM^42^ (average 24‐hour heart rate of <65 bpm); (3) sinus node dysfunction (dogs with and without clinical signs).

At least 1 board‐certified veterinary cardiologist from each institution made the diagnosis with a review to confirm (NSM). No cardiac medications, myocardial failure, or medications that affect heart rate were permitted for the balanced autonomic modulation and sinus node dysfunction dogs. Sotalol was the only drug permitted in the HP/LSM dogs used for testing. A diagnosis of sinus node dysfunction included consideration of the following parameters: heart rate (<65 bpm), minimum heart rate (<35 bpm), pauses >2 s (>3000), pauses >4 s (>5), and longest pause ≥5.5 s. These characteristics were made based on reported values for normal dogs.^23^, ^43^ It was not required that all these features be met, instead that the diagnostic assessment included consideration of these values in the decision process (eg, a dog with sinus node dysfunction could have >200 pauses >5.5 s, but average heart rate >65 bpm because of tachy‐brady rhythm). Careful attention was made to exclude any recordings that could represent examples of vasovagal response or accentuated antagonism.^20^, ^44^, ^45^ Vasovagal response was defined as <3 pauses/24‐hours >4 s that were preceded by a gradual slowing of the heart rate coupled with no other indicators of sinus node dysfunction. Accentuated antagonism was diagnosed as <3 pauses/24‐hours >4 s that were preceded by rapid sinus tachycardia and motion artifact compatible with excitement or exercise and no other characteristics of sinus node dysfunction. All non‐sinus escape complexes were deleted from the RR interval file because only sinus beats were used for analysis. The purpose was to permit the determination of the number and duration of sinus pauses and the beat‐to‐beat patterns of sinus‐initiated complexes identified in the RR interval data files. By eliminating the escape beats when no sinus P waves were present, the true duration of a sinus pause was determined.

Only recordings were included with sinus node dysfunction that exhibited the hypothesized exit block pattern of beat interval “clustering” as identified in the ECG, Poincaré plots, and tachograms.^25^, ^43^ Determination of whether or not the atrial depolarization was the result of initiation within the sinus node and exit from different sinoatrial pathways, macro‐reentry involving the sinus node complex or atrial ectopic foci was not possible; however, the goal was to select beat‐to‐beat clustering that would be compatible with a pattern of hypothesized exit block. Consequently, the identified clustering was reflective of intervals that approximated multiples of the prevailing shortest intervals.^25^ The criteria used in humans of sinus arrest or block based on the duration of a pause relative to the preceding intervals^46^ was not applicable.

Finally, correctness in beat annotation was essential, therefore, editing for >99% accuracy was required for the balanced autonomic modulation and HP/LSM recordings, and >95% accuracy was set for the sinus node dysfunction recordings. The lower level of precision was permitted because of the time required for editing some records of sinus node dysfunction. The time‐consuming process to correctly classify beats during a 24‐hour recording is established.^47^, ^48^

### 2.2 Variables determined from Holter recordings

Heart rate data over the 24‐hours (average heart rate, average RR interval, minimum heart rate, time <50 bpm, number of pauses >2 s, >3 s, and >4 s, and longest sinus pause) were recorded. Average heart rate, average RR interval, minimum heart rate and time <50 bpm were collected during stable/sleep hours. Stable/sleep hours were selected based on the 1 to 6 hours between 2200 and 0700 hours whereby the heart rate was lowest, the number of pauses >2 s was highest and the visual examination of the tachogram indicated pattern stability. Previous evaluation (results in Figure S1) of heart rate variability in control dogs and dogs with sinus node dysfunction showed that time domain parameters of heart rate variability track similarly (Giacomazzi F, Pariaut R, Santilli R, Moïse NS. Exit block as a mechanism of sinus node dysfunction evidenced by geometric heart rate variability. J Vet Int Med 2017; 31 (4)). Consequently, a single parameter was selected to avoid multiple comparisons; the heart rate corrected root mean square of successive differences between normal RR intervals (cRMSSD) was determined during 24‐hours and the stable/sleep hours.^49^, ^50^, ^51^, ^52^

### 2.3 Tachograms and Poincaré plots

Tachograms and Poincaré plots were created using software previously reported.^25^ RR interval data was exported from the Trillium software and uploaded to the open‐access website: https://www.thenextheartbeat.com/.

Quantification of differences in the Poincaré plots amongst the 3 diagnoses was undertaken by measurements of the linear changes in heart rate: (1) shortest interval value on the line of identity, (2) longest interval value on the line of identity, (3) range of beat‐to‐beat cluster on the line of identity. To quantify the minimum bifurcation interval representing a divergence from the line of identity,^23^ the shortest value of the horizontal cluster on the y‐axis (RR +1 interval) was measured. Qualitatively, observations were noted for the identified patterns of beat‐to‐beat intervals with comparisons to the ECG. During the collection of the Holter recordings showing the beat clustering on the tachograms and Poincaré plots, 2 “overall” patterns were noted with the sinus node dysfunction recordings. For simplicity, these were identified as 1 and 2. Additional comparisons of the heart rate data and the Poincaré plots were made between these 2 data sets.

### 2.4 Machine learning and Poincaré density grid

**FIGURE 2:**
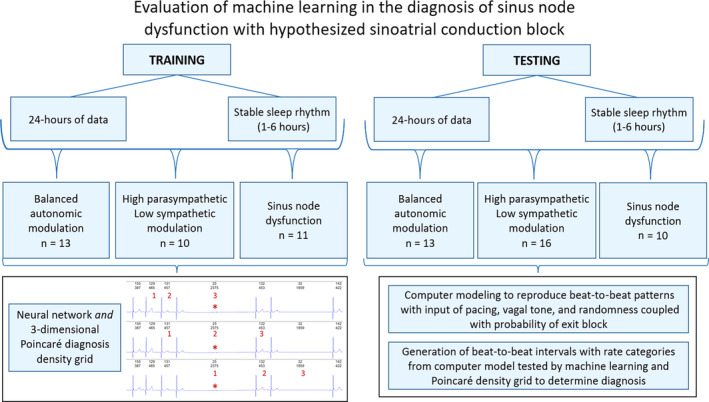
Flowchart used for the training and testing of machine learning to differentiate dogs with balanced autonomic modulation, HP/LSM, and sinus node dysfunction. To evaluate the model supporting sinoatrial conduction block the computer model generated intervals that also were tested with the neural network and the Poincaré density diagnosis grid. A 3‐beat interval relationship was used for the supervised training and then testing. Each interval was classified into 1 of the 3 diagnoses after it was judged in the first, second, and third interval location of a rolling interval appraisal. If judged differently during the 3 interval shift an average diagnosis was made. Therefore, every interval was categorized.

The study design is summarized in Figure 2. Data from dogs with each of the 3 diagnoses were randomly assigned to either the training or testing set using a random number generator. The 6 dogs treated with sotalol were included only in the test set.

Supervised machine learning was applied to the beat‐to‐beat intervals exported as a text file from the 24‐hour data plus the stable/sleep hours. The training and testing of the neural network were implemented in JavaScript (Pluralsight, Jerusalem, Israel). Three categories were trained for differentiation: balanced autonomic modulation, HP/LSM, and sinus node dysfunction. A 3‐layer (input layer, hidden layer, output layer) neural network with rectified linear (ReLu) activation function and a multiple frequency sine mapping was used. The neural network was initialized with random values. The loss function was *x* ^2^. The network was trained using gradient descent. For each diagnosis 3 beat‐to‐beat intervals were used for training and testing (Figure 2). Based on these 3 intervals (4 QRS complexes) each interval was classified by its relationship to 3 other intervals.

A beat‐to‐beat density grid was created from the training set because the premise of the study is that between the 3 diagnoses, distinctive beat‐to‐beat patterns exist. Therefore, a 3‐dimensional plot of all labeled beat intervals based on the diagnosis was collectively plotted in space (>3.5 million intervals). These data were used to create a diagnostic grid to evaluate the testing data sets. Based on the beat‐to‐beat interval relationships, each interval of the testing set was placed within a cell that had been categorized into the 3 diagnoses.

An open‐access website was created for the machine learning (http://comparepoincare.com/Classifier/machinelearning.html) and the density grid (http://comparepoincare.com/Classifier/grid.html). The beat‐to‐beat interval test files were uploaded for classification based on the results of the training set. The diagnosis/classification of each beat‐to‐beat interval on the tachograms and Poincaré plots was represented by 1 of 3 colors for each of the 3 diagnoses (balanced autonomic modulation, green; HP/LSM, blue; sinus node dysfunction, red). Finally, a decision based on machine learning and Poincaré density grid was shown by naming the diagnosis with the corresponding color code. The “relative sureness” of the decision for each method was displayed by the relative size of the words.

### 2.5 Computer modeling of exit block

The computer simulation model (Matlab R2021a, Mathworks, Natick, MA) consisted of 2 components: (1) the internal clock, which generates beats within the sinus node, and (2) the external beat generator, which determines which of the beats generated by the internal clock are blocked, and which exit. This model defined the time intervals between the impulses generated by the pacing cells of the sinus node, and whether an impulse was conducted or blocked was determined by its rate (the oscillator), variations in parasympathetic tone such as that from breathing, the probability of exit block, the rate at which the probability changes, and the influence of previous impulses blocking.

The time intervals between the beats generated by the internal clock are governed by the parasympathetic (vagal) modulation, which is assumed a function of the time t(in ms):

$$\text{vagal}\_\text{tone}\left(t\right)=0.08+\frac{1}{8}\left[1+\sin\left(\frac{2\mathrm{\pi t}}{4000}\right)\right]+\frac{3}{8}\left[1-\tanh\left(\cos(\frac{3}{2}\pi \frac{t}{\left(24000\right)\left(3000\right)},)\right)\right].$$

The first term is a small offset (0.08) because parasympathetic modulation is always present at a low level. The second term is the 4 s variation in vagal tone corresponds to a respiratory rate of 15 breaths/minute. The third term, which rises and falls slowly over 24 hours, is meant to mimic the circadian wake‐sleep cycle. The time intervals, $T_{int}$, are then defined through the equation,

$$T_{\mathrm{int}}=T_{0}+\text{vtdps}*\text{vagal}\_\text{tone}\left(t\right)+T_{\mathrm{rand}}*\mathrm{rand},$$

where vtdps, the vagal‐tone‐dependent probability sensitivity, was set to 400, $T_{\mathrm{rand}}=40$ if the previous beat was not blocked, and to $T_{b}$ (defined below) if it was, and rand is a random number chosen uniformly between 0 and 1. This definition for the time intervals is based on the reasonable assumption that the heart will beat at some rapid interval ($T_{0}$) in the absence of parasympathetic effects, and that this interval will lengthen as the parasympathetic tone is increased. The random term is meant to represent other effects lying outside the scope of the model.

The second component of the model is defined through the probability that any given beat generated by the first component of the model (ie, the internal clock) is blocked as it tries to exit the sinus node. This probability is also dependent on the parasympathetic modulation:

$$p_{\text{block}}=\left\{\begin{array}{cc} 0 & \text{if}\;\text{vagal}\_\text{tone}<v_{\mathrm{th}}\\ \text{ebpsf}*\left(\text{vagal}\_\text{tone}-v_{\mathrm{th}}\right) & \text{if}\;\text{vagal}\_\text{tone}>v_{\mathrm{th}} \end{array},\right.$$

where $v_{\mathrm{th}}=0.6$. Above this threshold, the *ebpsf*, the exit block probability scale factor, determines the rate at which block probability increases with parasympathetic tone.

To model the normal dog, $T_{0}=200,T_{b}=200$ and ebpsf=0.7 were set. To model the HP/LSM dog, $T_{0}=500$ was set to account for the inherently slower beat interval of these dogs. For the sinus‐node dysfunction dog model, $T_{0}=200,T_{b}=50$, and ebpsf=1.8 were set. The ebpsf parameter was set to a higher value for sinus node dysfunction to model the disproportionally strong effect of dysfunction at higher parasympathetic levels.

### 2.6 Statistical evaluation

Statistics were performed using JMP, Version *<17>* (SAS Institute Inc., Cary, North Carolina). Data are presented with medians and the 25th and 75th quartiles. The nonparametric statistics tests, Kruskal‐Wallis test, and a post hoc Wilcoxon test was performed to determine which pairs of groups had significant differences (*P* < .05). A 3 × 3 confusion matrix was created for the 24‐hour data set of the actual diagnosis and predictive diagnosis outcomes of machine learning and the Poincaré plot diagnosis density grid. Additionally, a 3 × 3 confusion matrix was constructed for the stable/sleep hour data of the 24‐hour diagnosis and predictive diagnosis. The confusion matrix was to show the accuracy of the actual to the predictive diagnosis with the ideal being complete agreement.

## 3 RESULTS

For balanced autonomic modulation, 26 recordings were included (total under review 137). For HP/LSM, 26 recordings were included (total under review 36). For sinus node dysfunction with a pattern that suggested sinoatrial conduction block, 21 recordings were included (total under review 92). This ratio of inclusion/exclusion cannot be used to evaluate the frequency of these diagnoses because other criteria could exclude the recording and not all recordings were available for review from the different institutions.

**FIGURE 3:**
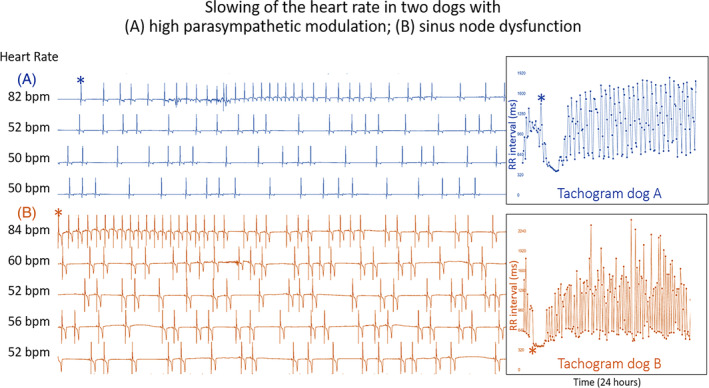
Continuous ECGs from Holter recording from 2 dogs. Tachograms overlay part of the recordings with the asterisk indicating the beginning of the time series that corresponds to the beat indicated with an asterisk on the ECG. Note how the heart rate slows following a brief interval of sinus tachycardia and the beat‐to‐beat patterns seen on the ECGs and the tachograms. (A) Normal Golden retriever with average 24‐hour heart rate of 57 bpm. (B) West Highland white terrier with sick sinus syndrome with average 24‐hour heart rate of 50 bpm. The pattern difference in the beat‐to‐beat intervals identified on the ECG can more clearly be identified on the tachograms. ms, milliseconds.

**FIGURE 4:**
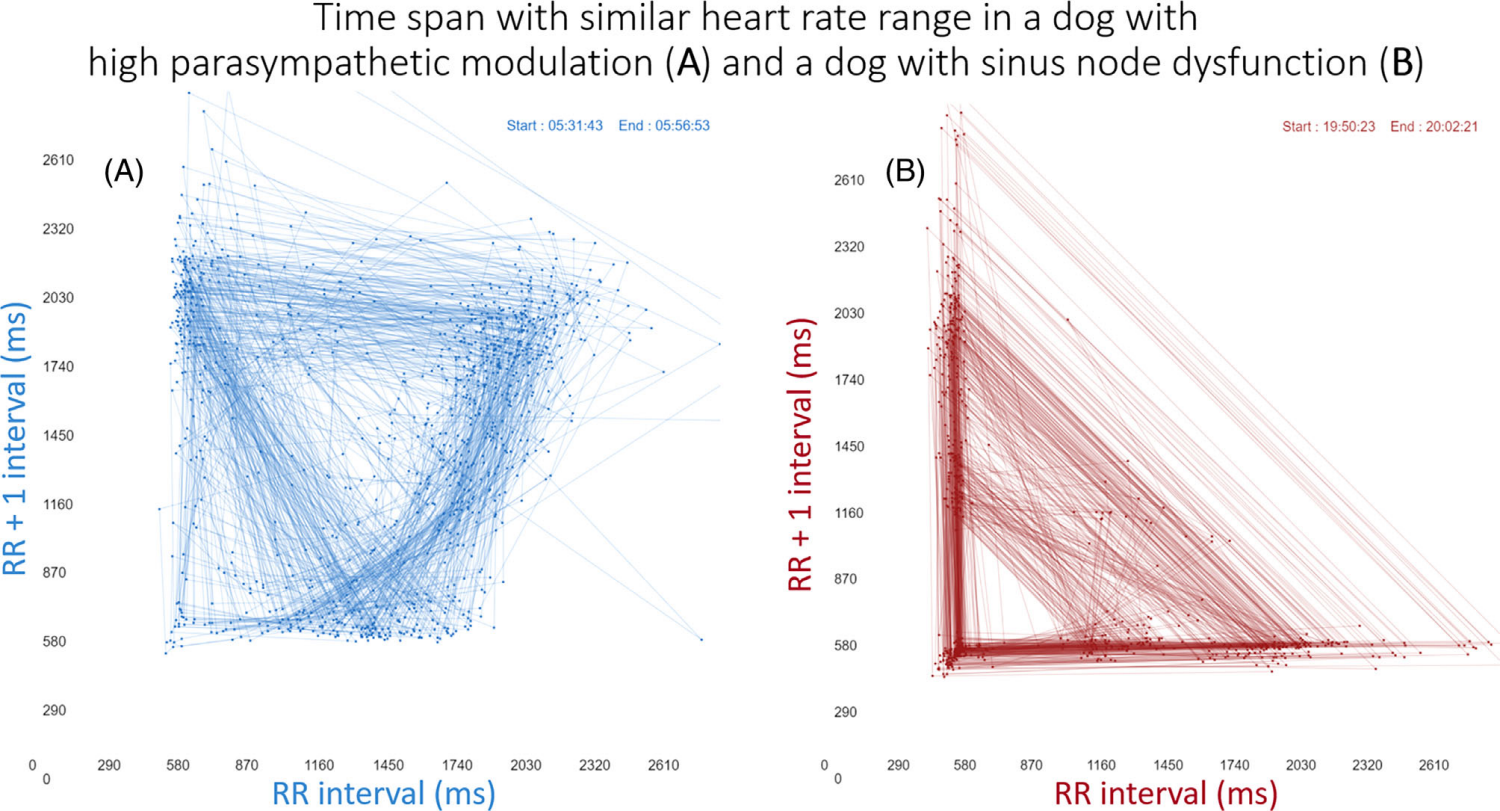
Poincaré plots of the same dogs shown in Figure 3. These time‐selected (upper right region of plots) Poincaré plots clearly demonstrate the difference in the beat‐to‐beat pattern in a dog with HP/LSM compared with a dog with sinus node dysfunction despite having similar heart rates. ms, milliseconds. See Video S1 to compare dynamic beat‐to‐beat Poincaré plots.

The signalment of the dogs studied and the reason for the Holter recording are shown Tables S1 and S2. Figure 3 illustrates an example of the time‐selected evaluations of the electrocardiograms and tachograms from a dog with HP/LSM compared with a dog with sinus node dysfunction. The differences in the beat‐to‐beat pattern between the HP/LSM and sinus node dysfunction recordings were evident in the Poincaré plots as shown in Figure 4 and Video S1.

Tables 1 and 2 provide the median and interquartile ranges and statistical evaluation for 24‐hour and stable/sleep hour variables for all recordings. To summarize, all heart rate variables for the balanced autonomic modulation recordings were significantly different from those of HP/LSM and sinus node dysfunction. The heart rate, RR interval, and duration of time that heart rate was <50 bpm in 24‐hours or minutes/hour (latter for stable/sleep hours) were not different between the HP/LSM and sinus node dysfunction recordings. However, the minimum heart rate, number of pauses (>2, >3, and >4 s) and longest pause duration were all significantly different (see Tables 1 and 2). These results were consistent for both the 24‐hours and the stable/sleep hours (number of pauses not evaluated for latter). The index of heart rate variability corrected for heart rate, cRMSSD, was significantly different for sinus node dysfunction when compared with both balanced autonomic modulation and HP/LSM dogs.

**TABLE 1:** The 24‐hour heart rate data (median 25th and 75th interquartile) and corrected RMSSD for dogs studied.

Variable	Balanced autonomics, n = 26	High parasympathetic and/or low sympathetic modulation, n = 26	Sinus node dysfunction, n = 21	Kruskal‐Wallis test, *P*‐value	Each pair Wilcoxon method, *P*‐value
Average HR (bpm)	81 (77‐92)	61 (57‐63)	62 (52‐73)	<.0001	<.0001^a^ <.0001^b^ .20^c^
Average RR interval (ms)	741 (650‐779)	984 (949‐1053)	968 (822‐1154)	<.0001	<.0001^a^ <.0001^b^ .20^c^
Minimum HR (bpm)	44 (41‐47.5)	32.5 (30.8‐34.3)	26 (24‐29)	<.0001	<.0001^a^ <.0001^b^ <.0001^c^
Time <50 bpm (min)	4.3 (0.1‐30.3)	594 (281.4‐723)	468 (180‐691)	<.0001	<.0001^a^ <.0001^b^ .10^c^
Number of pauses >2 s	44.5 (15.8‐202)	2340.5 (583‐3947)	8503 (7078‐10 050)	<.0001	<.0001^a^ <.0001^b^ <.0001^c^
Number of pauses >3 s	0 (0‐1.3)	50.5 (0.8‐123.8)	2199 (1047.5‐4219)	<.0001	<.0001^a^ <.0001^b^ <.0001^c^
Number of pauses >4 s	0 (0‐0)	1 (0‐9.3) (1 do824)	217 (58‐576.5)	<.0001	<.0001^a^ <.0001^b^ <.0001^c^
Longest pause (s)	2.7 (2.45‐3.3)	4.1 (2.9‐4.63)	6.9 (5.5‐10.3)	<.0001	.0003^a^ <.0001^b^ <.0001^c^
cRMSSD (ms)	0.445 (0.399‐0.485)	0.478 (0.440‐0.558)	0.787 (0.733‐0.930)	<.0001	.41^a^ <.0001^b^ <.0001^c^

Abbreviations: bpm, beats per minute; cRMSSD, heart rate corrected root mean square of successive differences between normal RR intervals; HR, heart rate; ms, milliseconds; s, seconds.

^a^ Balanced autonomic modulation compared with high parasympathetic and/or low sympathetic modulation.

^b^ Balanced autonomic modulation compared with sinus node dysfunction.

^c^ Sinus node dysfunction compared with high parasympathetic and/or low sympathetic modulation.

**TABLE 2:** Stable sleep‐hour heart rate data (median 25th and 75th interquartile) and corrected RMSSD for dogs studied.

Variable	Balanced autonomics, n = 26	High parasympathetic and/or low sympathetic modulation, n = 26	Sinus node dysfunction, n = 21	Kruskal‐Wallis test, *P*‐value	Each pair Wilcoxin method, *P*‐value
Average HR (bpm)	64 (58.8‐67)	48.5 (45.8‐51)	45 (41.5‐54)	<.0001	<.0001^a^ <.0001^b^ .45^c^
Average RR interval (ms)	937 (895‐1021)	1237 (1176‐1311)	1333 (1111‐1447)	<.0001	<.0001^a^ <.0001^b^ .45^c^
Minimum HR (bpm)	46 (41‐50)	33.5 (31.8‐36)	26 (23.5‐28.5)	<.0001	<.0001^a^ <.0001^b^ <.0001^c^
Time <50 bpm (min/hour)	0.3 (0‐4.8)	47.5 (39.8‐53.7)	42 (16.5‐53)	<.0001	<.0001^a^ <.0001^b^ .42^c^
cRMSSD (ms)	0.520 (0.471‐0.533)	0.502 (0.437‐0.549)	0.799 (0.657‐0.877)	<.0001	1.0^a^ <.0001^b^ <.0001^c^

Abbreviations: bpm, beats per minute; cRMSSD, heart rate corrected root mean square of successive differences between normal RR intervals; HR, heart rate; ms, milliseconds.

^a^ Balanced autonomic modulation compared with high parasympathetic and/or low sympathetic modulation.

^b^ Balanced autonomic modulation compared with sinus node dysfunction.

^c^ Sinus node dysfunction compared with high parasympathetic and/or low sympathetic modulation.

**FIGURE 5:**
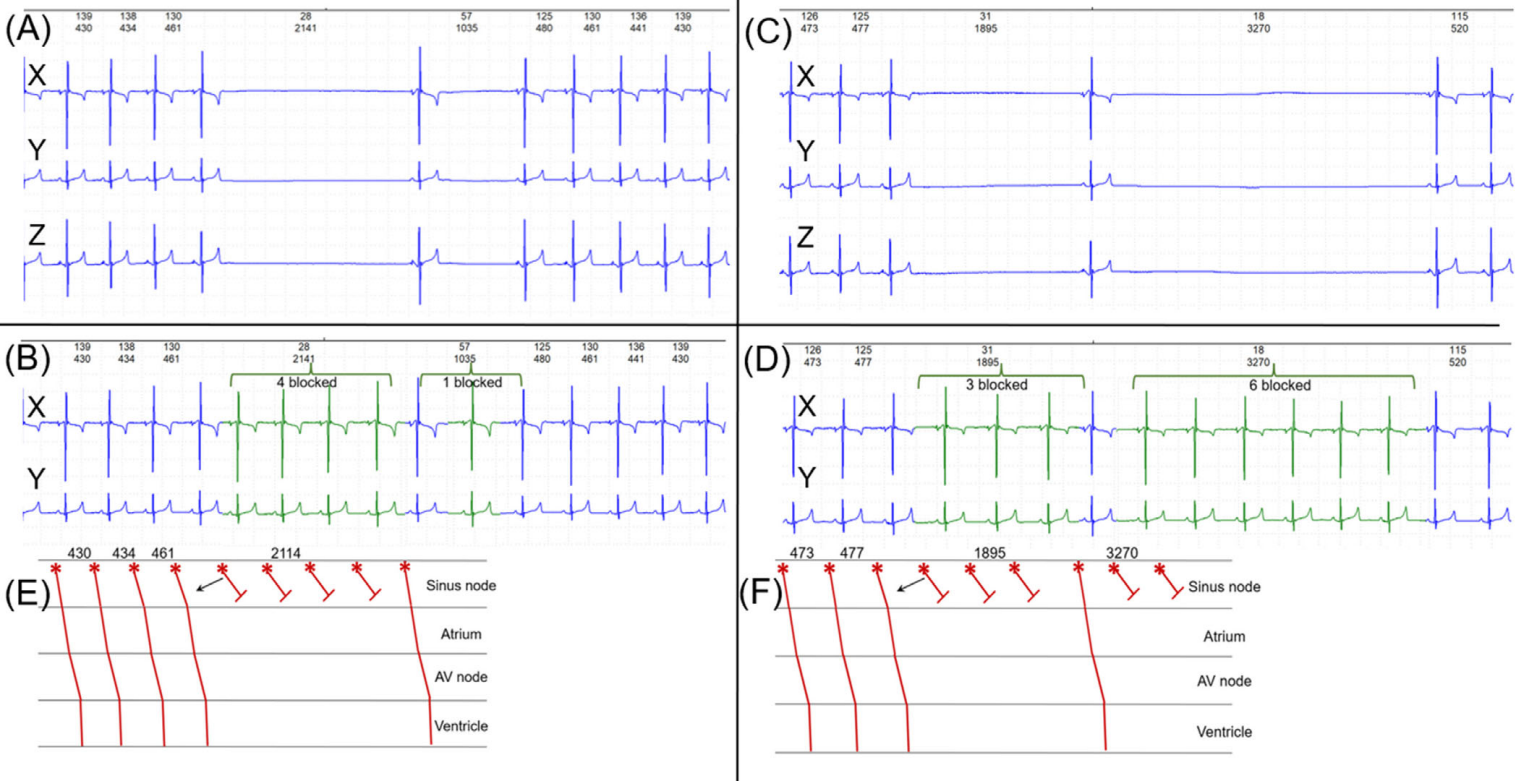
ECG (leads X, Y, and Z) frames from a dog with sinus node dysfunction. The pattern is not typical of sinus arrhythmia. Sinus node pauses were evident throughout the entire 24‐hours of the recording. Frame A and frame C show pauses that support a diagnosis of sinus node exit block. In frames B and D the copied QRS complexes (shown in green) and intervals that just precede the blocks are “inserted” into the pauses to illustrate the regularity and ratios of block. In frame E a laddergram corresponds to the portion of the ECG just above in frame B. (The 3rd lead is omitted in this frame for clarity.) The laddergram illustrates a potential mechanism for the pauses. The rate, although not a tachycardia, may be fast enough to result in decremental conduction in diseased sinoatrial conduction pathways. The arrow points to the line representing first delayed conduction (Type 1 Wenckebach sinoatrial block)^53^ because of earlier impulse initiation causing hypothesized decremental conduction from the sinus node and advanced sinoatrial block. Similarly illustrated in Frame F.

**FIGURE 6:**
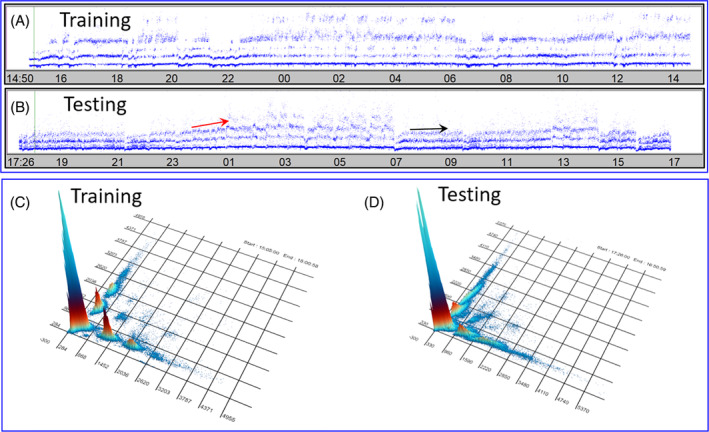
Examples of tachograms and 3‐dimensional Poincaré plots from 24‐hour Holter recordings of dogs diagnosed with sinus node dysfunction that displayed a pattern of grouping of RR intervals. This type of pattern in the tachograms (A and B) showed multiple “blurry” lines of beat intervals with a paucity of intervals between. Variation in the specific patterns is evident; however, a distinct difference exists when compared with normal dogs.^23^, ^25^ The red arrow shown highlights the change in the RR intervals such that with slowing of the heart rate, the RR interval relationship persists but with longer beat‐to‐beat intervals during the stable/sleep hours compared with the more constant relationship (black arrow) during the waking period giving evidence of the effects of increased parasympathetic modulation either on pacing cell oscillation, conduction velocity in sinoatrial conduction pathways or both. The Poincaré plots that correspond to the tachograms are shown in C and D. Additional examples shown in S2 and S3. Tachograms: *x*‐axis 24‐hour clock, *y*‐axis RR interval range 100 to 6000 ms. Poincaré plots: RR interval: *x*‐axis (ms). RR +1 interval: *y*‐axis (ms).

An example of an electrocardiogram consistent with exit block is shown in Figure 5. Without high‐resolution optical mapping^3^, ^4^, ^17^, ^21^ it is not possible to confirm a diagnosis of sinoatrial conduction block; however, the laddergrams shown in Figure 5 illustrate the plausibility.^53^ The tachograms and Poincaré plots from dogs with sinus node dysfunction are shown in Figures 6, S2, and S3 with additional mechanisms contributing to the duration of pauses shown in Figure S4.

**FIGURE 7:**
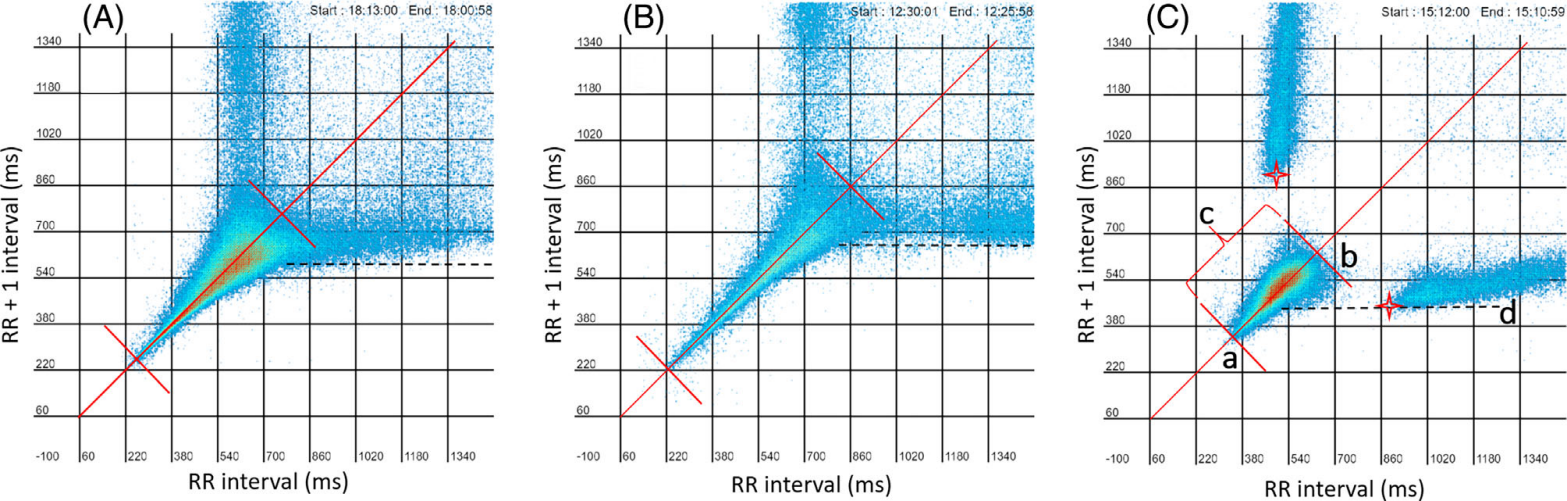
Measurements of Poincaré plots for (A) balanced autonomic modulation, (B) HP/LSM, and (C) sinus node dysfunction are shown in these 3 examples. The following were measured: a—shortest interval value line of identity, b—longest interval value line of identity, c—Range of cluster on line of identity, d—Shortest value *y*‐axis of deviation from the line of identity. Note the differences in the shape and beat‐to‐beat distribution (scales are equal for comparisons) which was quantified (measurements in Table 6). Additionally, note the relatively high density of intervals in the center of the line of identity for the sinus node dysfunction dogs. This distribution indicated that although the intervals did not reach the same maximum heart rates as the other dogs, short intervals were still prevailing.

Quantification of the beat‐to‐beat heart rate changes that occurred along the line of identity on the Poincaré plots are shown in Figure 7. These examples illustrate the results of the measurements reported in Table 3. To summarize, the range of beat‐to‐beat intervals in dogs with sinus node dysfunction was smaller than the other diagnoses. That is, the heart rate in these dogs did not go as fast as dogs with balanced autonomic modulation or HP/LSM, but the bifurcation from the line of identity occurred at faster rates. The longest range of beat‐to‐beat heart rate along the line of identity was present in the HP/LSM recordings.

**TABLE 3:** Beat‐to‐beat cluster data (median 25th and 75th interquartile).

Variable	Balanced autonomics, n = 26	High parasympathetic and/or low sympathetic modulation, n = 26	Sinus node dysfunction, n = 21	Kruskal‐Wallis test, *P*‐value	Each pair Wilcoxon method, *P*‐value
Shortest interval on line of identity (ms)	265 (239‐280)	260 (251‐292)	315 (278‐323)	<.0008	<.45^a^ .0001^b^ .008^c^
Longest interval on line of identity (ms)	720 (699‐754)	843 (799‐888)	620 (565‐698)	<.0001	<.0001^a^ <.0002^b^ <.0001^c^
Range of intervals on line of identity (ms)	463 (424‐518)	568 (533‐621)	345 (258‐390)	<.0001	<.0001^a^ <.0001^b^ <.0001^c^
Shortest horizontal deviation (ms)	575 (521‐605)	640 (600‐664)	425 (360‐465)	<.0001	.0002^a^ <.0001^b^ <.0001^c^

Abbreviations: bpm, beats per minute; HR, heart rate; ms, milliseconds.

^a^ Balanced autonomic modulation compared with high parasympathetic and/or low sympathetic modulation.

^b^ Balanced autonomic modulation compared with sinus node dysfunction.

^c^ Sinus node dysfunction compared with high parasympathetic and/or low sympathetic modulation.

**FIGURE 8:**
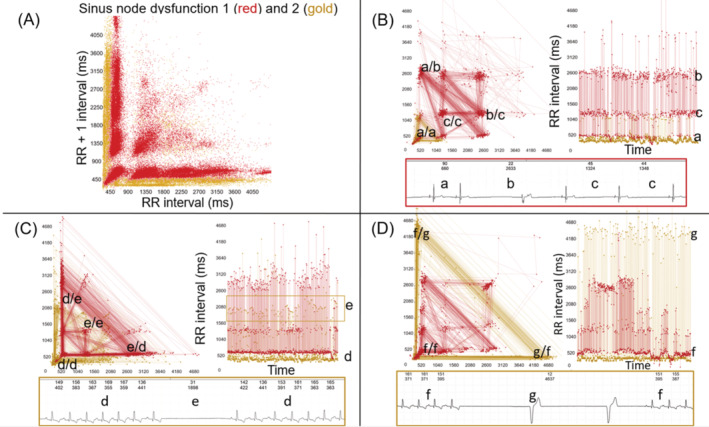
Still frames of the Poincaré plots from 2 variations in the exit block pattern of sinus node dysfunction. (A) Five dogs with sinus node dysfunction‐1 (red) are overlaid with 5 dogs with sinus node dysfunction‐2 (gold). The beat‐to‐beat intervals have similar clusters amongst the 5‐sinus node dysfunction‐1 dogs as seen by the overlay showing regions with no beat‐to‐beat intervals (arrow shows example). This indicates that although approximately a half‐million beat‐to‐beat intervals were plotted, similar clustering resulted in a paucity of intervals in the same region. Such a relationship suggests a similar mechanism governing the complexity of sinus node behavior. Sinus node dysfunction‐2 dogs had shorter beat‐to‐beat intervals than sinus node dysfunction‐1 dogs and clustering characterized by shorter intervals associated with long intervals. The latter caused the “arms” of the Poincaré plot to align more parallel to the axes than dogs with sinus node dysfunction‐1. (B‐D) Poincaré plots, tachograms and ECGs are shown from a dog with sinus node dysfunction‐1 (red) and a dog with sinus node dysfunction‐2 (gold). The data from both dogs are overlaid for comparisons. The color of the box surrounding the ECG corresponds to the color of the specific diagnosis. The ECG intervals are labeled with the duration in milliseconds indicated with the instantaneous heart rate shown above. The corresponding position on the Poincaré plot and tachograms are shown with the corresponding lower‐case letters. The RR interval, which is a surrogate for the PP interval on the Poincaré plot, appears as the first letter separated from the RR +1 interval by a slash (eg, a/b). (B) The ECG within the red box shows the intervals that create the positions on the Poincaré plot and tachogram. Note the consistent clustering of the beat‐to‐beat intervals that is characteristic of an exit block as supported by the beat‐to‐beat intervals approximating multiples of the shortest interval. Interval “a” is 660 ms (90 bpm), interval “b” is 2633 ms (22 bpm) which is ~660 ms × 4 = 2640 and interval “c” averages 1336 ms (44 bpm) which is ~660 ms × 2 = 1320. (C) Although the intervals are different in the dog with sinus node dysfunction‐2 (gold) than those noted for the dog with sinus node dysfunction −1 (red), clustering is still evident (note gold box outlining the gold dots plotted in the tachogram with a lowercase “e”). The average of the intervals identified as “e” in the ECG, Poincaré plot and tachogram is 4.9 (~5) × the average of the intervals identified as “d” ~ 390 ms (154 bpm) × 5 = 1950 ms (31 bpm). (D) The interval duration noted as “g” is ~12 × 380 ms (158 bpm) = 4560 ms (13 bpm). The “tightness” of the clusters (see C and D) diminishes with longer intervals which is an expected mathematical phenomenon. None of the escape beat intervals are included in the ECG durations and the plots. Retrograde atrioventricular conduction with depolarization of the atria is seen in the escape complexes. None could be associated with alteration in the sinus node frequency. Axes for Poincare plots shown in (A). bpm, beats per minute; ms, milliseconds. See Video S2.

Two‐ and 3‐dimensional Poincaré plots and tachograms suggested a general variation in the type of beat‐to‐beat interval clustering in the dogs with sinus node dysfunction. By observation, the type of clustering was categorized and equal distribution between training and testing data. For simplicity, the categories of sinus node dysfunction‐1 and sinus node dysfunction‐2 were used. Sinus node dysfunction‐1 had more distinct separation of clusters, as the RR interval increased the corresponding RR +1 interval increased resulting in a cluster that commonly deviated up and to the right on the Poincaré plot. Furthermore, recordings described as sinus node dysfunction‐1 displayed a cluster with a long‐long interval along the line of identity. Sinus node dysfunction‐2 had less distinct clusters, the RR interval vs RR +1 interval more closely aligned with the *x*‐ and *y*‐axes. A comparison of these 2 types of exit block patterns is shown in Figure 8. The complexity of the potential mechanisms (parasympathetic modulation with Wenckebach conduction block and decremental conduction) leading to a sinus pause because of intranodal or sinoatrial conduction pathway block is shown in Figure S4.

Measurements (Table 4) of the Poincaré plot clusters revealed that sinus node dysfunction‐2 had a smaller‐longest interval on the line of identity compared with sinus node dysfunction‐1. This resulted in a significantly shorter range of beat‐to‐beat intervals on the line of identity, and, therefore, a more compact cluster. Also, the bifurcation from the line of identity occurred at shorter beat‐to‐beat intervals for sinus node dysfunction‐2 vs 1. The latter finding suggests that a beat‐to‐beat relationships of shorter‐short intervals and longer‐long intervals in sinus node dysfunction‐2. This is visually appreciated in Figure 8. The only heart rate and rhythm parameter that differed between the 2 types of sinus node dysfunction was a higher cRMSSD in sinus node dysfunction‐2.

**TABLE 4:** Beat‐to‐beat cluster data, heart rate, and RMSSD for sinus node dysfunction 1 and 2 [median (25th and 75th interquartile)].

Variable	Sinus node dysfunction 1, n = 11	Sinus node dysfunction 2, n = 10	Kruskal‐Wallis test, *P*‐value
Shortest interval on line of identity (ms)	315 (300‐320)	303 (273‐336)	.75
Longest interval on line of identity (ms)	690 (668‐735)	565 (315‐605)	.0003
Range of intervals on line of identity (ms)	385 (345‐435)	258 (195‐296)	.0006
Shortest horizontal deviation (ms)	450 (425‐485)	360 (308‐399)	.002
24‐hour HR (bpm)	61 (50‐68)	68 (60‐85)	.08
Minimum HR (bpm)	26 (21‐28)	27 (24‐29)	.38
Number of pauses >2 s	7548 (306‐1008)	9486 (111‐512)	.39
Number of pauses >3 s	1222 (669‐3002)	3514 (1653‐5188)	.05
Number of pauses >4 s	185 (53‐547)	308 (59‐2327)	.36
Longest pause (s)	6.4 (5.5‐9.9)	7.8 (5.5‐10.9)	.60
24‐hour cRMSSD (ms)	0.750 (0.648‐0.783)	0.930 (0.879‐1.022)	.0007

Abbreviations: bpm, beats per minute; cRMSSD, heart rate corrected root mean square of successive differences between normal RR intervals; HR, heart rate; ms, milliseconds; s, seconds.

### 3.1 Machine learning and beat‐to‐beat density grid

**FIGURE 9:**
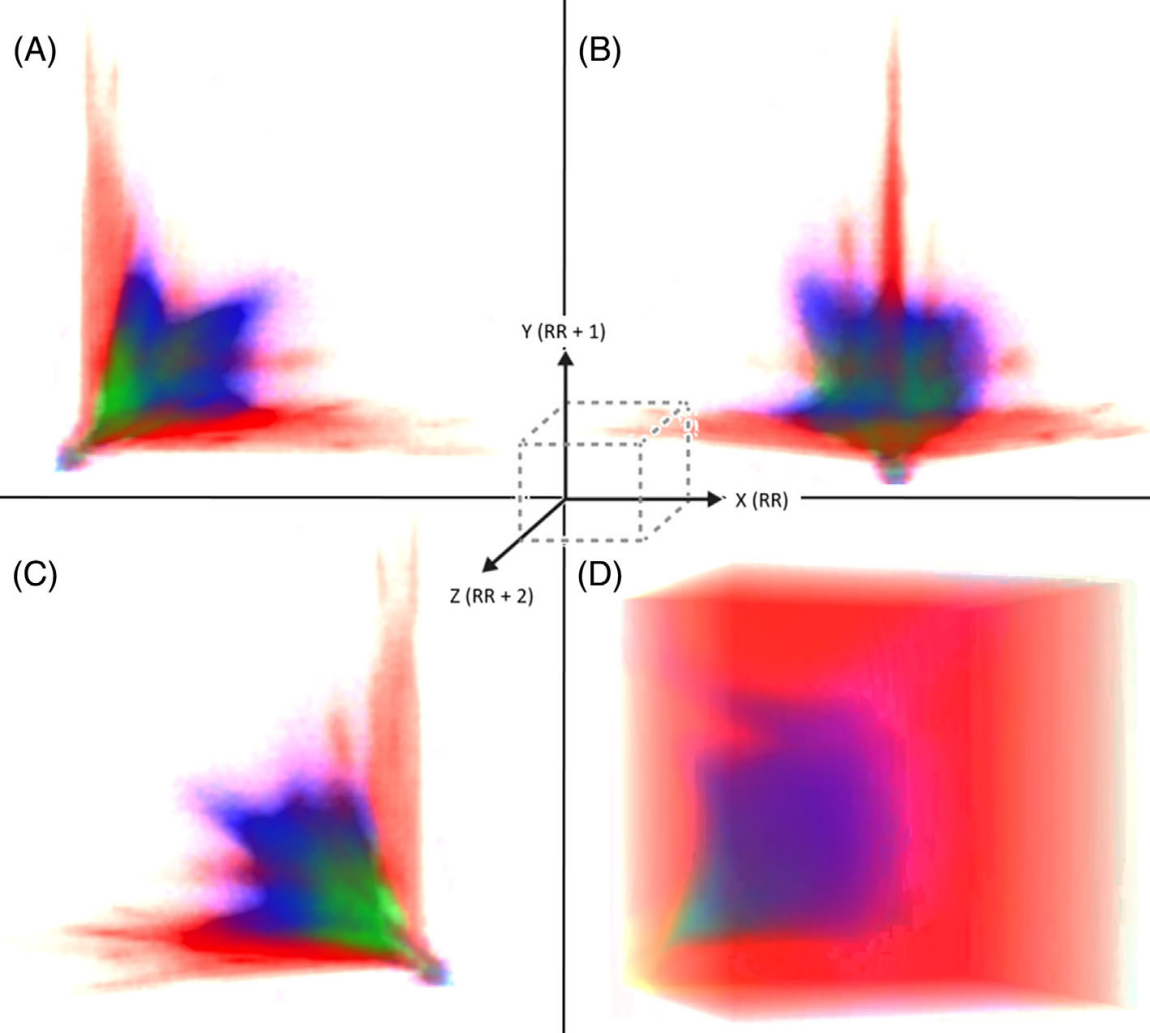
From the training data (composed of >3.5 million intervals see Figure S5) a grid was created for the classification of testing data sets. Frames A, B, and C are still frames from the dynamic 3‐dimensional dynamic Poincaré plots of this grid. Within this grid 1 million cells were created into which a test beat‐to‐beat interval could “fall” based on the training interval position. Each cell of the grid is 60 ms × 60 ms × 60 ms. The total time domain in each plane was 6000 ms. Poincaré diagnostic density grid requires each beat‐to‐beat interval to fall within a cell, and, therefore, no “decision” will be made if outside of the cells of the map. Consequently, the grid does not make assumptions as compensatory machine learning. Frame D shows a still frame from 3‐dimensional (*x*, *y*, *z* plot) dynamic map created from the neural network (machine learning) trained dataset for each of the 3 categories: balanced autonomic modulation, HP/LSM, and sinus node dysfunction. Three beat intervals were mapped to multiple sine frequencies followed by a 2‐layer perceptron (neural network) run. This plot gives all possible outputs of the neural network. For the machine learning diagnosis, assumptions (guessing) based on “learning” can be made for the beat‐to‐beat intervals. Green represents the balanced autonomic modulation; blue represents HP/LSM; and red represents sinus node dysfunction. Fused colors (eg, shades of purple) indicate regions of mixed categories (Videos S3 and S4). ms, milliseconds.

From the beat‐to‐beat intervals uploaded from each of the 3 test data sets, machine learning and the Poincaré density grid classified all intervals (Figure 2). Figure S5 shows the actual overlay in 3‐dimensional space of all beat‐to‐beat intervals of the training data from which the Poincaré density grid and machine learning were created (Figure 9). The confusion matrix comparing the actual 24‐hour beat‐to‐beat diagnosis to that made by machine learning and the Poincaré density grid are shown in Tables 5 and 6. The confusion matrix comparing the 24‐hour diagnosis to that made by machine learning and the Poincaré density grid for the stable/sleep hours are shown in Tables 7 and 8.

**TABLE 5:** The 24‐hour heart rate data for randomly assigned for training vs testing [median (interquartile values)].

Parameter	Balanced autonomics		High parasympathetic and/or low sympathetic modulation		Sinus node dysfunction		*P* value
	Training, n = 13	Testing, n = 13	Training, n = 10	Testing, n = 16	Training, n = 11	Testing, n = 10	
Average HR (bpm)	84 (77.5‐92.5)	79 (76‐89)	61 (57‐64)	61 (57.5‐62)	67 (59‐84)	61.5 (51.5‐68.5)	NS
Average RR interval (ms)	714 (649‐774)	759 (675‐789)	984 (938‐1053)	984 (968‐1044)	895 (714‐1017)	976 (876‐1165)	NS
Minimum HR (bpm)	45 (42‐52)	43 (41‐46)	32.5 (30.8‐35.3)	32.5 (29.5‐34)	28 (24‐30)	25.5 (22‐26.5)	NS
Time <50 bpm (min)	1.9 (0‐26.1)	13.8 (1.0‐55.7)	567.5 (346.5‐798)	606 (516‐700.5)	318 (132‐674)	532 (270‐783)	NS
Number of pauses >2 s	41 (6‐80)	45 (27‐215)	3281 (477‐5738)	1921 (743‐3716)	7972 (6317‐12 000)	9010 (7091‐9998)	NS
Number of pauses >3 s	0 (0‐1.5)	0 (0‐2.5)	23 (0‐156.3)	67 (1.5‐109.8)	1668 (412‐4463)	2948 (1211‐4255)	NS
Number of pauses >4 s	0 (0‐0)	0 (0‐0)	0 (0‐4.5)	0 (0.3‐14.5)	158 (48‐198)	330 (68‐575)	NS
Longest pause (s)	2.7 (2.2‐3.4)	2.8 (2.6‐3.3)	3.5 (2.8‐4.4)	4.3 (3.2‐5)	6.3 (5.5‐7.9)	8.4 (5.5‐12.2)	NS
RMSSD (ms)	0.459 (0.418‐0.506)	0.426 (0.397‐0.467)	0.507 (0.448‐0.621)	0.470 (0.413‐0.506)	0.787 (0.733‐0.938)	0.780 (0.712‐0.932)	NS

**TABLE 6:** The 24‐hour confusion matrix comparing *Poincaré plot density diagnosis grid* predictive to actual diagnosis.

		Actual diagnosis		
		Balanced autonomic modulation	+Parasympathetic−sympathetic modulation	Sinus node dysfunction
Predictive	Balanced autonomic modulation	13	3	0
	+Parasympathetic−sympathetic modulation	0	13	0
	Sinus node dysfunction	0	0	10
	Total number	13	16	10

**TABLE 7:** Stable sleep‐hours confusion matrix comparing *machine learning* prediction to 24‐hour diagnosis.

		Diagnosis based on 24‐hour category		
		Balanced autonomic modulation	+Parasympathetic−sympathetic modulation	Sinus node dysfunction
Predictive	Balanced autonomic modulation	4	1	0
	+Parasympathetic−sympathetic modulation	9	15	0
	Sinus node dysfunction	0	0	10
	Total number	13	16	10

**TABLE 8:** Stable sleep‐hours confusion matrix comparing *Poincaré plot density diagnosis grid* prediction to 24‐hour diagnosis.

		Diagnosis based on 24‐hour category		
		Balanced autonomic modulation	+Parasympathetic−sympathetic modulation	Sinus node dysfunction
Predictive	Balanced autonomic modulation	9	1	0
	+Parasympathetic−sympathetic modulation	4	15	0
	Sinus node dysfunction	0	0	10
	Total number	13	16	10

**FIGURE 10:**
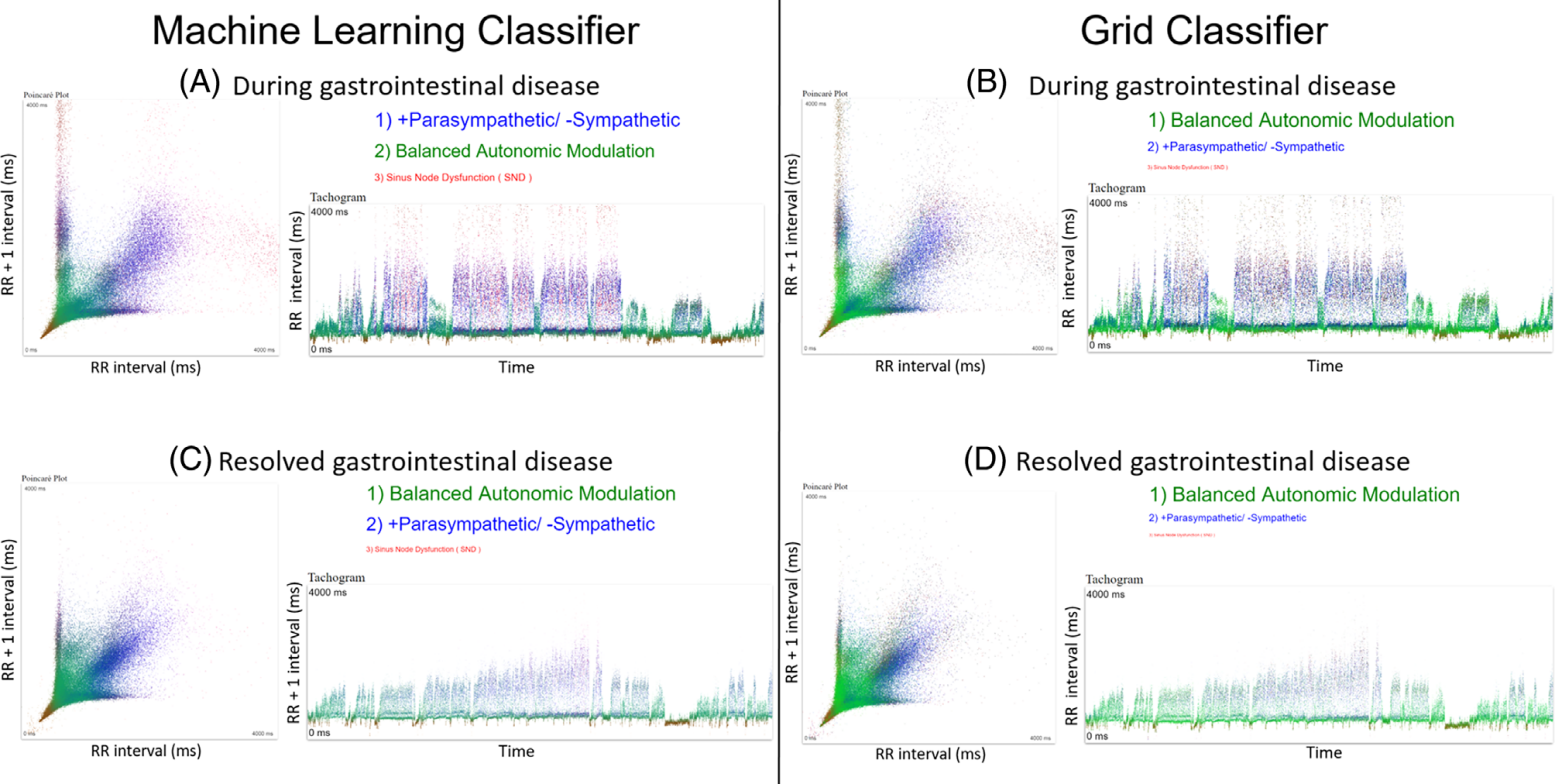
Example of the classification results of machine learning and the grid classifier of a Holter recording from a dog classified with discordant results. Two 24‐hour Holter recordings were made 3‐months apart. Initially, during a gastrointestinal illness a Holter recording revealed a heart rate of 57 bpm. Machine learning (A) diagnosed HP/LSM and the grid classifier (B) diagnosed balanced autonomic modulation. More of a difference in the individual beat interval diagnosis is seen with the color‐coding. The relative size of the letters indicates the relative level of indecision for the other 2 diagnoses. The words and dots have the same color‐coding (green, balanced autonomic modulation; blue, HP/LSM, and red, sinus node dysfunction). The follow‐up Holter recordings revealed an average 24‐hour heart rate of 74 bpm. Both methods diagnosed a balanced autonomic modulation; however, notice the relative differences between the 2 methods by the size of the diagnosis words. bpm, beats per minute; ms, milliseconds.

**FIGURE 11:**
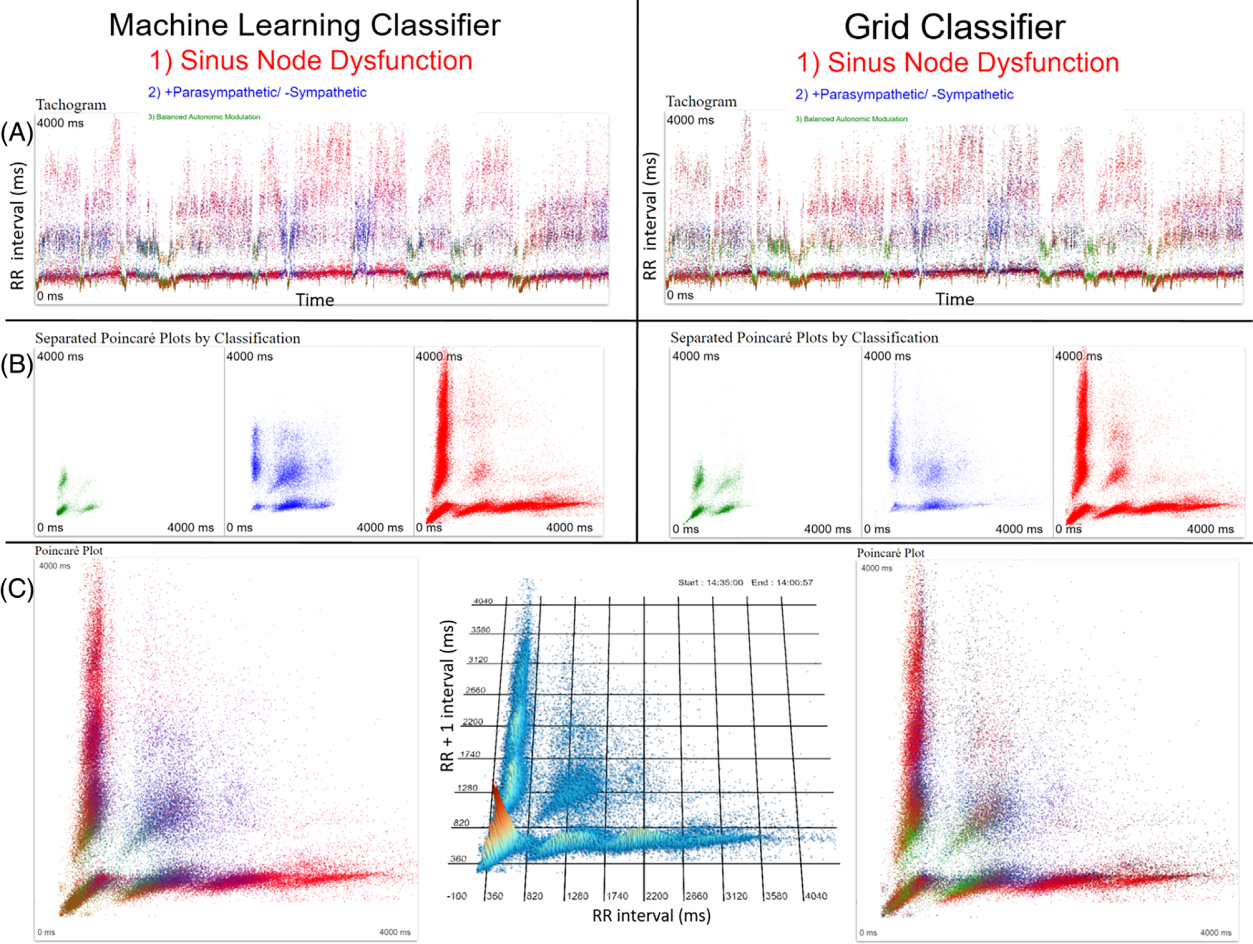
Example of the classification results of machine learning and the grid classifier of a Holter recording from a dog classified with sinus node dysfunction‐1. Both methods made the correct diagnosis ranking sinus node dysfunction #1. The relative size of the letters indicates the level of indecision for the other 2 diagnoses. The words and dots have the same color‐coding (green, balanced autonomic modulation; blue, HP/LSM, and red, sinus node dysfunction). The color‐coding of the dots in the tachograms (A) show how each method identified the intervals. Intervals for which the diagnosis was mixed appear as a mixed color (eg, purple for red and blue). Similarly, the Poincaré plots (B) under the tachograms show the distribution by the beat‐to‐beat determination that is then summarized in (C) with the 24‐hour Poincaré plot with all beat‐to‐beat intervals overlaid. The 3‐dimensional plot in the center of frame C shows the beat density of the 24‐hour results. Note for clarity the axes for the Poincaré plots is only indicated on this center image. Note the modest differences in the interval classification (B). ms, milliseconds.

**FIGURE 12:**
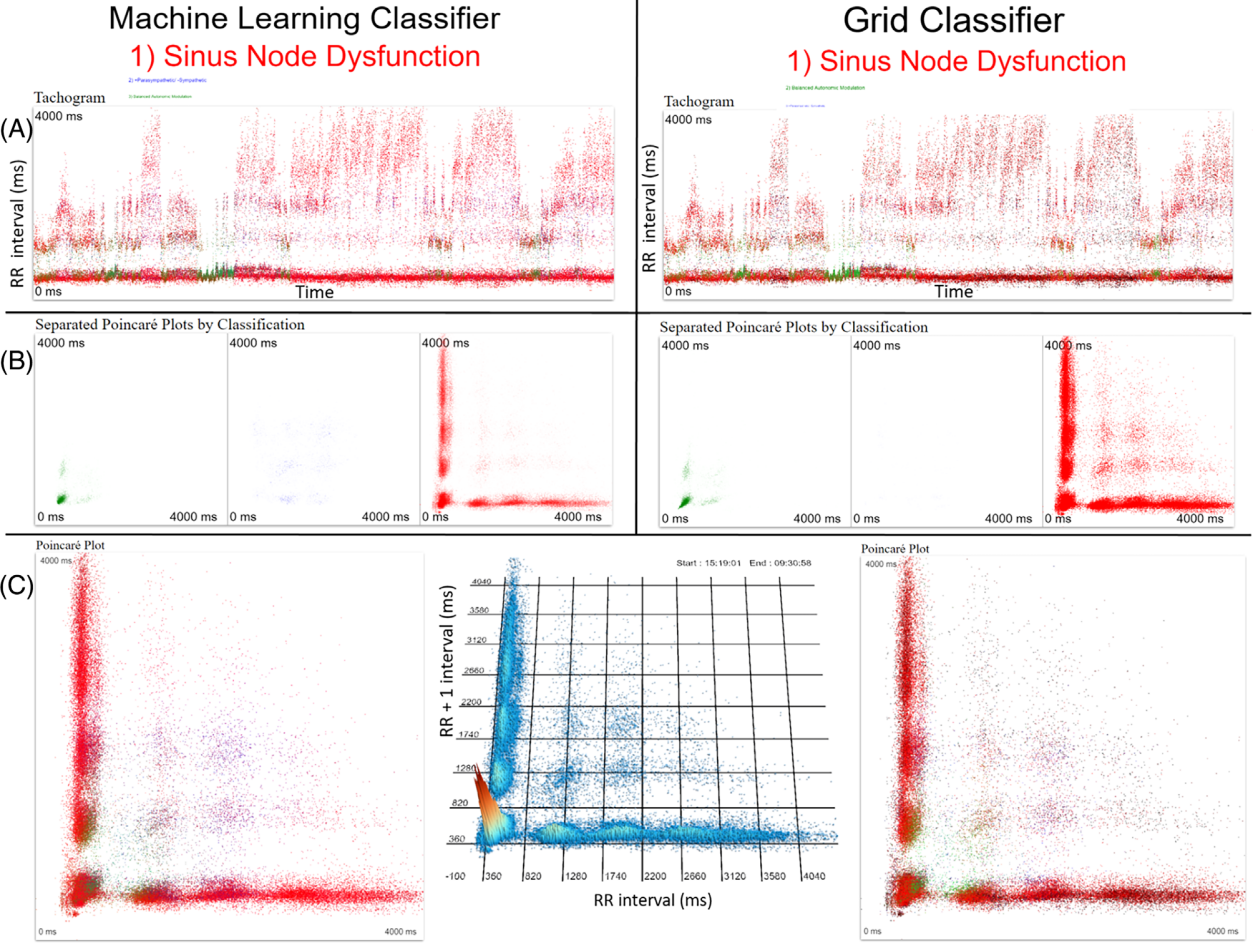
Example of the classification results of machine learning and the grid classifier of a Holter recording from a dog classified with sinus node dysfunction‐2. Both methods made the correct diagnosis ranking sinus node dysfunction #1. The relative size of the letters indicates the level of indecision for the other 2 diagnoses. The words and dots have the same color‐coding (green, balanced autonomic modulation; blue, HP/LSM; and red, sinus node dysfunction). The color‐coding of the dots in the tachograms (A) show how each method identified the intervals. Intervals for which the diagnosis was mixed appear as a mixed color (eg, purple for red and blue). Similarly, the Poincaré plots (B) under the tachograms show the distribution by the beat‐to‐beat interval determination that is then summarized in the 24‐hour Poincaré plot shown in (C) with all beat‐to‐beat intervals overlaid. The 3‐dimensional plot in the center of frame C shows the beat density of the 24‐hour results. Little indecision for either method is identified. Note for clarity the axes for the Poincaré plots is only indicated on this center image. Note the modest differences in the interval classification (B). ms, milliseconds.

Figures S6 and S7 show examples of results for the machine learning and density grid for balanced autonomic modulation and HP/LSM, respectively. Figure 10 shows an example of a discordant result in a dog with gastrointestinal disease. Machine learning diagnosed HP/LSM while the density grid diagnosed balanced autonomic modulation. Following resolution of the gastrointestinal disease both methods were concordant with a diagnosis of balanced autonomic modulation. Comparisons of the 2 methods for sinus node dysfunction‐1 and 2 are shown in Figures 11 and 12.

### 3.2 Computer modeling of sinoatrial conduction block

The model that incorporated key components such as the base oscillation rate, variation in parasympathetic modulation and probability of exit block successfully generated intervals that reproduced the actual data and were correctly diagnosed by the machine learning and the Poincaré density grid classifier (Figures S8 and 13). Importantly, during the development of the model parameters, input variables were critical to reproduce the patterns of exit block. For example, the base oscillation rate of 520 ms for sinus node dysfunction was shorter than the 820 ms that reproduced the results for high parasympathetic/low sympathetic data. Moreover, the exit block probability scaling factor was higher for sinus node dysfunction. Thus, the computer model strongly supports the hypothesis that sinoatrial conduction block is responsible for the beat‐to‐beat patterns identified in these dogs with sinus node dysfunction.

**FIGURE 13:**
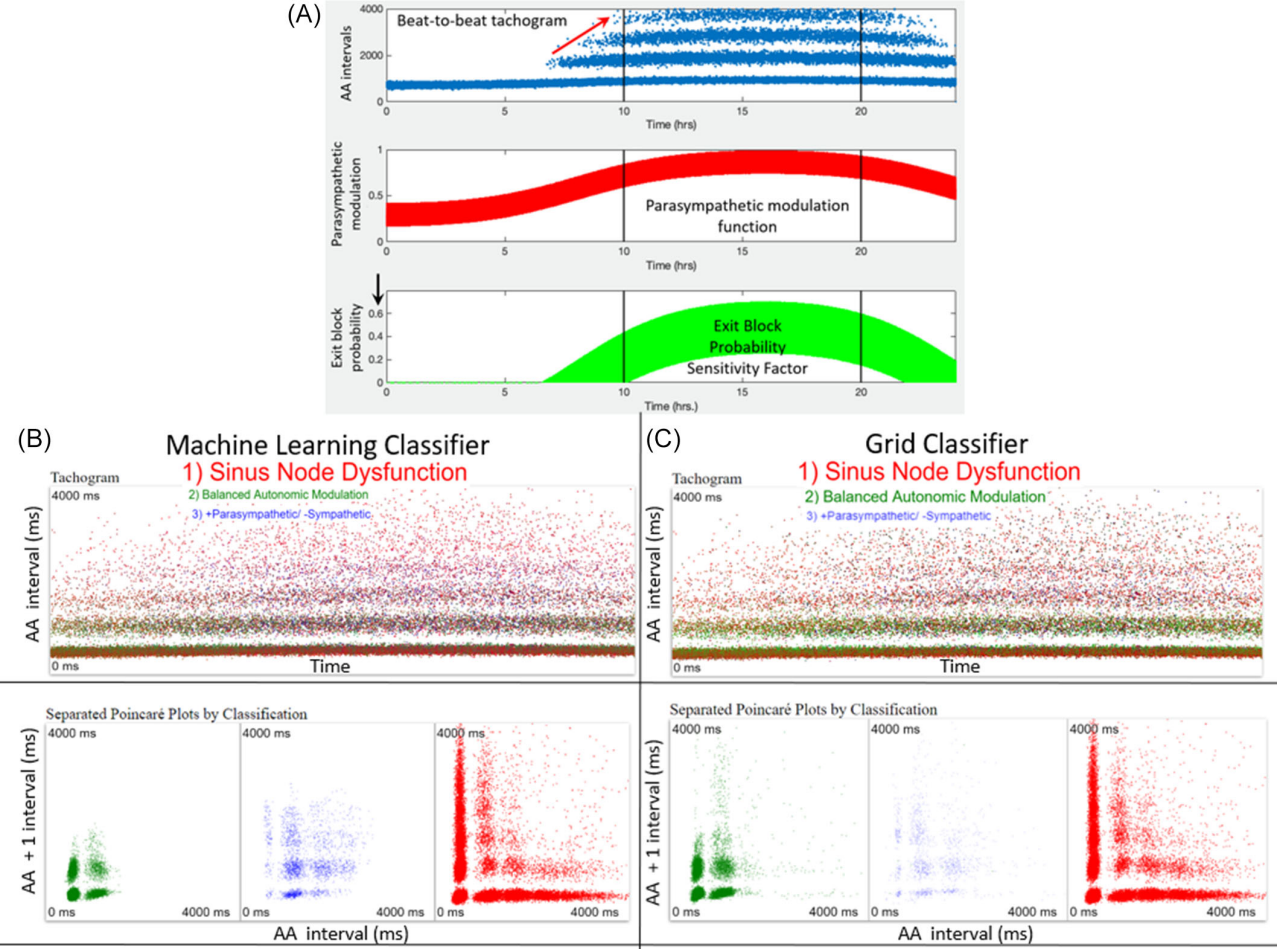
Computer modeling for exit block during conduction from the sinus node (the oscillator) to the atrial myocardium. In the model for sinus node dysfunction a base rate of 530 ms was used. This interval was derived as the average of those in the lowest band seen on the tachogram (A) which is changed by the increasing or decreasing parasympathetic modulation and a randomness factor. As parasympathetic modulation increases, so does the probability of exit block (A). Note the increasing number of bands with increased parasympathetic modulation (red arrow) and the higher value for the exit block probability in the sinus node dysfunction model compared with the HP/LSM model (Figure S8). The computer‐generated intervals during this time [demarcated by the vertical black lines in (A)] were then uploaded into the machine learning (B) and grid (C) classifiers. Note the differences in the classification, but the same final diagnosis. Green = balanced autonomic modulation, blue = HP/LSM, red = sinus node dysfunction. ms, milliseconds.

## 4 DISCUSSION

The major finding of this study is that machine learning algorithms and a Poincaré density grid could differentiate sinus node dysfunction with sinoatrial conduction block from HP/LSM and balanced autonomic modulation in dogs. Specific results indicate that: (1) variables of heart rate can differentiate HP/LSM from sinus node dysfunction; (2) quantitative measures of the interval patterns seen by electrocardiography, tachograms and Poincaré plots can differentiate sinus node dysfunction from those of HP/LSM and balanced autonomic modulation; (3) machine learning and Poincaré density grid methods have the ability to differentiate sinus node dysfunction from autonomic modulation; and (4) a computer simulation model supports the hypothesis that sinoatrial conduction block is responsible for the characteristic beat‐to‐beat patterns identified in this subset of dogs with sinus node dysfunction.

The dogs included in this study were those hypothesized to have sinoatrial conduction block based on the ECGs, tachograms and Poincaré plots.^4^, ^5^, ^6^, ^24^, ^28^, ^29^, ^53^, ^54^, ^55^ Notably, in the diseased sinus node, multiple overlapping mechanisms are possible for failed impulse generation and conduction. The anatomy and function of the sinus node is complex with profound influence on rate and rhythm from the autonomic nervous systems. Hypotheses for normal changes in rate and rhythm include different intrinsic pacing rates associated with a hierarchical pacemaker clustering of cells,^30^ dependence on functional autonomic innervation,^56^ existence of 2 sinus nodes^57^ and shifts in the exit of impulses through pathways.^6^, ^15^, ^58^ Although disease of the sinoatrial conduction pathways can cause dysfunction, sinus arrhythmia patterns in the normal dog are also consistent with blockade in these specialized pathways.^17^, ^23^ Nonetheless, extensive disease can advance the degree of exit block and lead to clinical signs. In humans and dogs, excessive fibrofatty infiltrate,^10^ increased acetylcholine,^21^ upregulation in heart failure of adenosine‐A1 receptors and GIRK mRNA expression,^14^, ^59^ and down regulation of hyperpolarization‐activated cyclic nucleotide‐gated channel protein expression (molecular components for *I*_*f*_ current) can cause slowed conduction and block that is intranodal or within the sinoatrial pathways.^16^ The beat‐to‐beat relationships, as measured herein on the ECGs, suggested both an increase in sinoatrial exit block under the influence of parasympathetic modulation and possibly decremental conduction within the sinoatrial conduction pathways.^3^ None of the dogs selected for this study had atrioventricular nodal block. In a recent study of the pathology of dogs with sinus node dysfunction, none had abnormalities of the atrioventricular node.^10^

Typically, bradycardia is a hallmark of sinus node dysfunction in dogs.^10^, ^11^, ^12^, ^20^, ^26^ However, the bradycardia identified in dogs that have HP/LSM associated with disease or breed differences may confound differentiation from dogs with sinus node dysfunction.^20^, ^60^ Reflecting this similarity, the 24‐hour average heart rate of sinus node dysfunction dogs in this study did not differ from that of dogs in the HP/LSM group. Additionally, the time that these 2 groups of dogs spend with heart rates <50 bpm was not different. Yet, although these heart rates were non‐discerning, what differentiated these dogs were specific characteristics of heart rate including the minimum heart rate, number of pauses (>2, >3, and >4 s) and duration of the longest pause. Although the signature feature of sinus node dysfunction is a slow heart rate, examination of the ECGs, tachograms and Poincaré plots revealed short beat‐to‐beat intervals (faster rates) followed by long sinus pauses. The substrate of slowed conduction that results in exit block also serves as a mechanism for reentry that is intranodal (micro‐reentry) or in the sinoatrial conduction pathways (macro‐reentry); each of which could result in the ECG rhythms of tachycardia‐bradycardia.^17^, ^18^, ^21^, ^30^

A single parameter for heart rate variability, cRMSSD, was selected as the measure for approximating parasympathetic mediated fluctuations.^61^, ^62^ When heart rate variability parameters are used, correction for heart rate is necessary.^49^, ^50^, ^51^, ^52^ Although the group of dogs defined as HP/LSM was based on a 24‐hour heart rate <65 bpm, the cRMSSD was not different from the group of dogs defined as balanced autonomic modulation. A possible explanation for this finding rests in the all‐pervading sinus arrhythmia and slowing of heart rate with increase in parasympathetic modulation during sleep reflected in healthy dogs as a heart rate nocturnal dip.^63^ That is, the dominance of parasympathetic modulation in the dog still prevails when the 24‐hour heart rate is between 74 and 96 bpm. This assessment also is supported by the number of dogs in the latter group classified by machine learning and Poincaré density grid as HP/LSM during the stable/sleep hours.

The cRMSSD in the sinus node dysfunction dogs was high. Although the influence of elevated parasympathetic modulation could contribute to this result, it is more likely to reflect sinus node disease^64^, ^65^, ^66^; known as heart rate fragmentation. Fragmented rhythms of sinus node dysfunction are characterized by a high percentage of reversals in heart rate acceleration causing the beat‐to‐beat relationship to be erratic.^65^, ^66^

Quantification of the Poincaré plots indicated that linear changes in heart rate were distinctly different amongst the 3 groups of dogs. Although the dogs with HP/LSM had significantly more linear slowing, these dogs were still capable of having rapid heart rates. Dogs with sinus node dysfunction were unable to obtain the same “shortest” beat‐to‐beat intervals and the linear range was smaller. The latter was not only because of the longer‐short intervals, but also the abrupt bifurcation with shorter‐long intervals on the line of identity. These findings are compatible with the development of sinoatrial conduction block at fast rates and yet, the diseased dogs had sinus nodes that were unable to go as fast as the normal dogs in response to high sympathetic modulation.^15^

Two sets of data from the sinus node dysfunction dogs displayed distinct differences in the Poincaré plots: likely indicating variation in mechanisms. Studies in the dog reveal that low concentrations of acetylcholine result in exit block in the sinoatrial conduction pathways, but higher concentrations additionally decrease spontaneous pacing rates because of suppression of phase 4 depolarization.^6^, ^21^ Moreover, faster rates can cause overdrive suppression of the sinus node pacemaker cells.^67^ Thus, these effects could hypothetically play a role in the different beat‐to‐beat relationships (Poincaré plot pattern) seen when comparing sinus node dysfunction‐1 and ‐2.

Supervised machine learning and Poincaré density grid creation provide a means to assist in the diagnosis of sinus node dysfunction particularly in differentiating bradycardias associated with HP/LSM. Because each interval is classified, insight is given to the decision that the algorithms made leading to the diagnostic conclusion. These 2 methods are different but yielded similar results. Machine learning permitted the algorithm to make assumptions based on “learning” such that when beat‐to‐beat intervals fell in regions on the 3‐dimensional plot not seen during training, a decision about identity was still made. Whereas the Poincaré density grid required that each beat‐to‐beat interval fall within a cell as determined during training. Thus, some differences in the final diagnosis are possible. However, in the confusion matrix the similarity in the results between the machine learning and Poincaré density grid is notable, although statistics were not performed because of the small number. Automatic recognition of cardiac arrhythmias and the geometric patterns seen with Poincaré plots has been reported^27^; however, within our report a specific disease in the dog was studied. Likely, these types of analyses will contribute extensively to clinical diagnostic ability.^33^, ^34^, ^35^, ^36^, ^37^, ^39^

The computer modeling supports the hypothesis that in dogs with sinus node dysfunction and beat‐to‐beat clustering, sinoatrial conduction block is a key mechanism. Previously, exit block has been hypothesized as a mechanism for the pattern of sinus arrhythmia in the dog.^23^ In the model, lower values of the exit block probability sensitivity created interval relationships of sinus arrhythmia, while high values created those of sinus node dysfunction. Deserving notation, in the model for sinus node dysfunction a shorter base interval in conjunction with increasing parasympathetic modulation led to greater exit block. This finding is concordant with the actual data showing that although dogs with sinus node dysfunction do not reach rates as fast as normal dogs, the deviation from linear rate slowing occurs abruptly at shorter intervals. Such findings beg the question: In dogs with sinus node dysfunction and patterns of sinoatrial conduction block; is this rhythm a tachycardia masquerading as a bradycardia?

## 5 LIMITATIONS

Machine learning in the diagnosis of long‐term ECG recordings is in its infancy and the methods must be sound to validate the results.^68^ Expanded deep learning will permit the recognition of the most discerning metrics in diagnosing rhythm disorders, thus offering more information than this current study.^68^ The data set used herein was small; however, it is meaningful that 2 different methods were used to make the diagnoses and they were meaningfully concordant. This study only evaluated dogs that were hypothesized to have sinoatrial conduction block. A computer simulated model was used to confirm the hypothesis of sinoatrial conduction block; however, actual high‐resolution mapping of the sinus node and sinoatrial conduction pathways would be required to prove this hypothesis.

## 6 CONCLUSIONS

In the dog, specific rate parameters, indications of heart rate fragmentation, quantifiable interval patterns identified on Poincaré plots, machine learning and Poincaré density grid algorithms and computer modeling enable the diagnosis of sinus node dysfunction with sinoatrial conduction block. Testing of additional data sets will provide expanded opportunities to evaluate the value of machine learning in the diagnosis of complex rhythm disorders.^68^ Moreover, detailed phenotyping of the beat‐to‐beat patterns with further model development could identify other mechanisms of sinus node dysfunction.

## CONFLICT OF INTEREST DECLARATION

Authors declare no conflict of interest.

## OFF‐LABEL ANTIMICROBIAL DECLARATION

Authors declare no off‐label use of antimicrobials.

## INSTITUTIONAL ANIMAL CARE AND USE COMMITTEE (IACUC) OR OTHER APPROVAL DECLARATION

Approval from CUVCSC and Exemption from IACUC Review, CUVCSC Protocol ID#: 090623‐12.

## HUMAN ETHICS APPROVAL DECLARATION

Authors declare human ethics approval was not needed for this study.

## Supporting information


**Figure S1.** Results of heart rate variability in control dogs and dogs with sinus node dysfunction. These preliminary results were presented as an oral abstract at the ACVIM meeting 2017 (Giacomazzi F, Pariaut R, Santilli R, Moise NS. Exit block as a mechanism of sinus node dysfunction evidenced by geometric heart rate variability. J Vet Int Med 2017; 31 [4]).
**Figure S2.** Additional tachograms from dogs with sinus node dysfunction and hypothesized sinoatrial conduction pathway block used to show similarity between the files of those trained and tested. The corresponding Poincare plots shown in Figure S3. Complements Figure 6.
**Figure S3.** Additional Poincaré plots from dogs with sinus node dysfunction and hypothesized sinoatrial conduction pathway block used to show similarity between the files of those trained and tested. The corresponding tachograms shown in Figure S2. Complements Figure 6.
**Figure S4.** The complexity of the potential mechanisms leading to a sinus pause because of conduction block (1st or 2nd degree) are suggested by the beat‐to‐beat interval relationships that can have opposing effects. Time‐selected tachogram (A) shows the relationship of beat interval clusters as the heart rate decreases (PP/RR intervals lengthens) and increases (PP/RR intervals shortens) with changes likely from autonomic modulation. As the shorter intervals increase (slowing heart rate) the next intervals slow to a greater extent (red arrow, red bars are the same length). This was observed in some dogs during the sleep hours (Figure 6B). It is not possible to quantify this observation because of the variation in the input that was determining the relationships. However, the observations illustrate the likely relationship of the beat‐to‐beat variability to autonomic influences. In contrast, Frame B1 and B2 are from the same time‐selected beat‐to‐beat intervals and illustrate as the short intervals (PP/RR intervals) get shorter (note arrows), the long intervals get longer which is the opposite effect of that shown in frame A. This would be consistent with decremental conduction in the sinoatrial conduction pathways. Additionally, the number of shorter intervals before the longer interval may have an effect [examine beat intervals before longest pause (c)]. Frame B2 shows the actual ECGs and intervals that are shown in B1 to the duration of the pauses.
**Figure S5.** Frames A, B, and C are from a dynamic 3‐dimensional Poincaré plot (*x* = RR interval, *y* = RR +1 interval, and *z* = RR +2) of all training data (~3.5 million intervals). Green represents the balanced autonomic modulation; blue represents HP/LSM and red represents sinus node dysfunction. Mixed colors (eg, purple or rust) represent intervals that overlay with a different classification (intervals with different diagnosis, but same location in 3‐dimensional space). See Video S4.
**Figure S6.** Example of the classification results of machine learning and the grid classifier of a Holter recording from a dog classified with a balanced autonomic modulation. Both methods made the correct diagnosis ranking balanced autonomic modulation #1. The relative size of the letters indicates the level of indecision for the other 2 diagnoses. The words and dots have the same color‐coding (green, balanced autonomic modulation; blue, HP/LSM, and red, sinus node dysfunction). The color‐coding of the dots in the tachograms (A) show how each method identified the intervals. Intervals for which the diagnosis was mixed appear as a mixed color (eg, brown for red and green). Similarly, the Poincaré plots (B) under the tachograms show the distribution by the beat‐to‐beat interval determination that is then summarized in the 24‐hour Poincaré plot shown in (C) with all beat‐to‐beat intervals overlaid. The 3‐dimensional plot in the center of frame C shows the beat density of the 24‐hour results. Note for clarity the axes for the Poincaré plots is only indicated on this center image. ms, milliseconds.
**Figure S7.** Example of the classification results of machine learning and the grid classifier of a Holter recording from a dog classified with a HP/LSM. Both methods made the correct diagnosis ranking HP/LSM #1. The relative size of the letters indicates the level of indecision for the other 2 diagnoses. The words and dots have the same color‐coding (green, balanced autonomic modulation; blue, high parasympathetic/ low sympathetic modulation, and red, sinus node dysfunction). The color‐coding of the dots in the tachograms (A) show how each method identified the intervals. Intervals for which the diagnosis was mixed appear as a mixed color (eg, purple for red and blue). Similarly, the Poincaré plots (B) under the tachograms show the distribution of the beat‐to‐beat interval determination that is then summarized in the 24‐hour Poincaré plot shown in (C) with all beat‐to‐beats overlaid. The 3‐dimensional plot in the center of frame C shows the beat density of the 24‐hour results. Note for clarity the axes for the Poincaré plots is only indicated on this center image. ms, milliseconds.
**Figure S8.** Computer modeling for exit block during conduction from the central sinus node to the atrial myocardium. In the model for high parasympathetic modulation a base rate of 830 ms was used. (A) This interval was derived as the average of those in the lowest band seen on the tachogram. Intervals change by the increasing or decreasing parasympathetic modulation (middle frame) and a randomness factor. As parasympathetic modulation increases, so does the probability of exit block. The exit block probability scale factor, (bottom frame) determines the rate at which block probability increases with parasympathetic tone. The computer‐generated intervals during this time (demarcated by the vertical black lines in (A) were then uploaded into the machine learning (B) and grid (C) classifiers). Note the modest differences in the beat‐to‐beat classification, but the same final diagnosis. Green = balanced autonomic modulation, blue = high parasympathetic modulation, red = sinus node dysfunction. ms, milliseconds.

**Table S1.** Descriptive data for dogs included in the study.
**Table S2.** Reason given for 24‐hour Holter performed.
**Table S3.** The 24‐hour heart rate data for randomly assigned for training vs testing [median (interquartile values)].

**Video S1.** Comparison of dynamic Poincaré plots from a dog with sinus node dysfunction (red) and a dog with high parasympathetic modulation (blue). Note beat‐to‐beat pattern differences during selected‐times when dogs had similar heart rates (300 beat‐to‐beat intervals shown at a time with a change of 100 beats/second). This video compliments Figure 4.
**Video S2.** Two different selected times during the 24‐hour recordings from 2 dogs overlaid for comparison. Dog in red has sinus node dysfunction‐1 and dog in gold has sinus node dysfunction‐2. (300 beat‐to‐beat intervals shown at a time with a change of 100 beats/second). This video compliments Figure 10.
**Video S3.** Video of 3‐dimensional dynamic map created from the neural network (machine learning) trained dataset. This plot gives all possible outputs of the neural network. Green represents the balanced autonomic modulation; blue represents HP/LSM and red represents sinus node dysfunction. RR = beat‐to‐beat interval. This video corresponds to Figure 12.
**Video S4.** Dynamic 3‐dimensional Poincaré plot of all training data (~3.5 million intervals). Green represents the balanced autonomic modulation, blue represents HP/LSM and red represents sinus node dysfunction. Mixed colors (eg, purple or rust) represent intervals that overlay with a different classification (intervals with different diagnosis, but same location in 3‐dimensional space). RR = beat‐to‐beat interval. This video corresponds to Figure 13.
