# Supplementary material for: Use of machine learning and Poincaré density grid in the diagnosis of sinus node dysfunction caused by sinoatrial conduction block in dogs
Source: J Vet Intern Med. 2024 Apr 29;38(3):1305–24. doi: 10.1111/jvim.17071 (PMC11099791; doi:10.1111/jvim.17071)
Supplement: Supplementary file 1 — Figure S1. Results of heart rate variability in control dogs and dogs with sinus node dysfunction. These preliminary results were presented as an oral abstract at the ACVIM meeting 2017 (Giacomazzi F, Pariaut R, Santilli R, Moise NS. Exit block as a mechanism of sinus node dysfunction evidenced by geometric heart rate variability. J Vet Int Med 2017; 31 [4]). Figure S2. Additional tachograms from dogs with sinus node dysfunction and hypothesized sinoatrial conduction pathway block used to show similarity between the files of those trained and tested. The corresponding Poincare plots shown in Figure S3. Complements Figure 6. Figure S3. Additional Poincaré plots from dogs with sinus node dysfunction and hypothesized sinoatrial conduction pathway block used to show similarity between the files of those trained and tested. The corresponding tachograms shown in Figure S2. Complements Figure 6. Figure S4. The complexity of the potential mechanisms leading to a sinus pause because of conduction block (1st or 2nd degree) are suggested by the beat‐to‐beat interval relationships that can have opposing effects. Time‐selected tachogram (A) shows the relationship of beat interval clusters as the heart rate decreases (PP/RR intervals lengthens) and increases (PP/RR intervals shortens) with changes likely from autonomic modulation. As the shorter intervals increase (slowing heart rate) the next intervals slow to a greater extent (red arrow, red bars are the same length). This was observed in some dogs during the sleep hours (Figure 6B). It is not possible to quantify this observation because of the variation in the input that was determining the relationships. However, the observations illustrate the likely relationship of the beat‐to‐beat variability to autonomic influences. In contrast, Frame B1 and B2 are from the same time‐selected beat‐to‐beat intervals and illustrate as the short intervals (PP/RR intervals) get shorter (note arrows), the long intervals get longer which is th [file JVIM-38-1305-s001.pdf]

Figure SI-1. Results of heart rate variability in control dogs and dogs with sinus node dysfunction. These preliminary results were presented as an oral abstract at the ACVIM meeting 2017. (Giacomazzi F, Pariaut R, Santilli R, Moise NS. Exit block as a mechanism of sinus node dysfunction evidenced by geometric heart rate variability.. J Vet Int Med 2017; 31 (4).)

Figure SI-1.

	Parameter	Median control dogs n = 24	Range control dogs	Median sinus node dysfunction dogs n= 24	Range sinus node dysfunction dogs	P-Value
TIME	SDNN (ms)	0.31	0.09 – 0.43	0.60	0.43 – 0.73	<0.0001
	SDANN (ms)	0.08	0.03 – 0.22	0.17	0.09 – 0.28	<0.0001
	RMSSD (ms)	0.4	0.07 – 0.60	0.94	0.57 – 1.30	<0.0001
FREQUENCY	LFnu	11.5	4 – 83	5	2 – 10	0.0007
	HFnu	88.5	17 – 96	95	90 – 98	0.0007
	LF/HF	0.13	0.04 – 5.05	0.05	0.02 – 0.11	0.0009

Figure SI-2. Additional tachograms from dogs with sinus node dysfunction and hypothesized sinoatrial conduction pathway block used to show similarity between the files of those trained and tested. The corresponding Poincare plots shown in Figure SI-3. Complements Figure 6.

Figure SI-2.

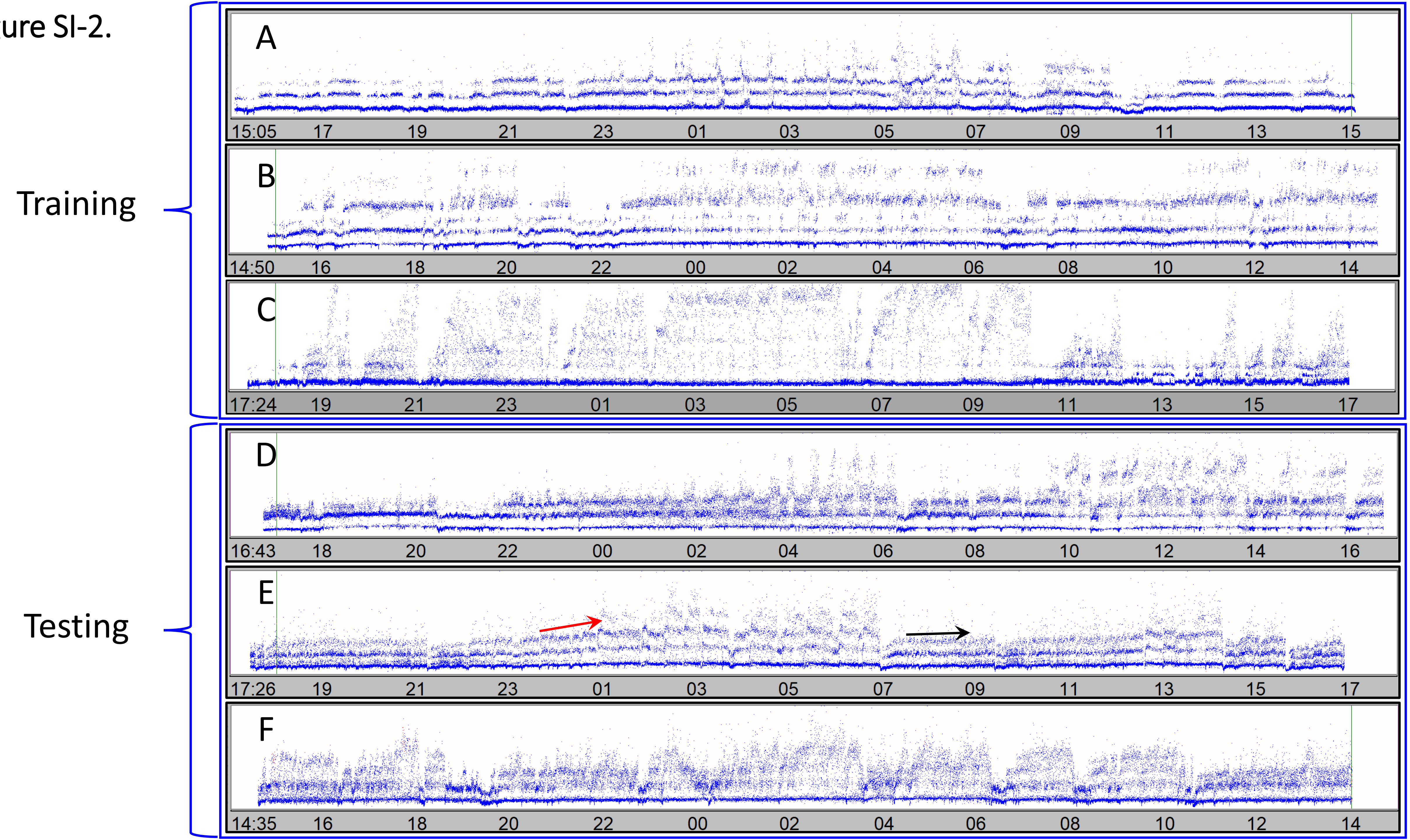
Training
Testing
A
15:05 17 19 21 23 01 03 05 07 09 11 13 15
B
14:50 16 18 20 22 00 02 04 06 08 10 12 14
C
17:24 19 21 23 01 03 05 07 09 11 13 15 17
D
16:43 18 20 22 00 02 04 06 08 10 12 14 16
E
17:26 19 21 23 01 03 05 07 09 11 13 15 17
F
14:35 16 18 20 22 00 02 04 06 08 10 12 14

Figure SI-3. Additional Poincaré plots from dogs with sinus node dysfunction and hypothesized sinoatrial conduction pathway block used to show similarity between the files of those trained and tested. The corresponding tachograms shown in Figure SI-2. Complements Figure 6.

Figure SI-3.

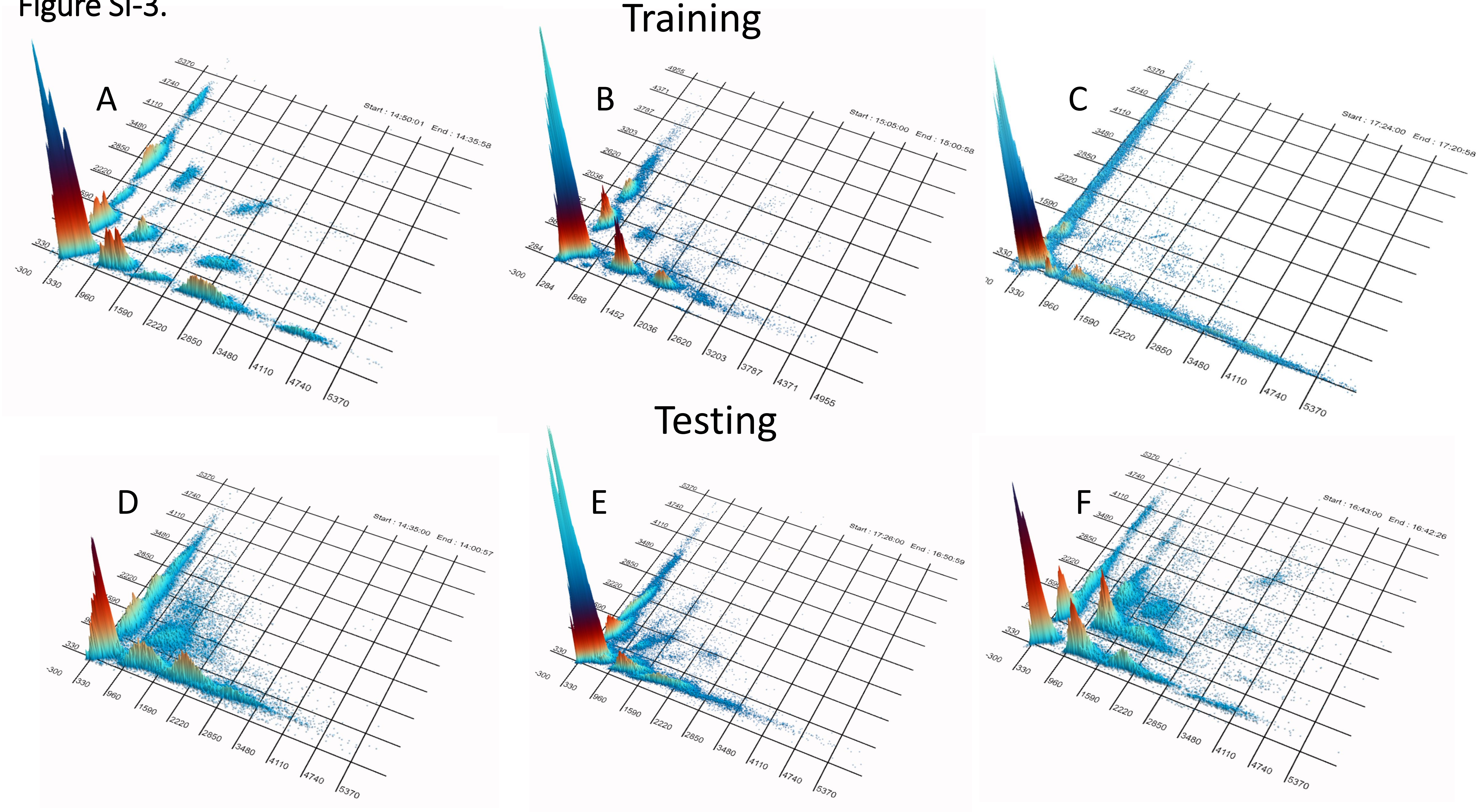
Training
A
Start : 14:50:01 End : 14:35:58
B
Start : 15:05:00 End : 15:00:58
C
Start : 17:24:00 End : 17:20:58
Testing
D
Start : 14:35:00 End : 14:00:57
E
Start : 17:26:00 End : 16:50:59
F
Start : 16:43:00 End : 16:42:26

Figure SI-4. The complexity of the potential mechanisms leading to a sinus pause because of conduction block (1st or 2nd degree) are suggested by the beat-to-beat interval relationships that can have opposing effects. Time-selected tachogram (A) shows the relationship of beat interval clusters as the heart rate decreases (PP/RR intervals lengthens) and increases (PP/RR intervals shortens) with changes likely from autonomic modulation. As the shorter intervals increase (slowing heart rate) the next intervals slow to a greater extent (red arrow, red bars are the same length). This was observed in some dogs during the sleep hours (Figure 6). It is not possible to quantify this observation because of the variation in the input that was determining the relationships. However, the observations illustrate the likely relationship of the beat-to-beat variability to autonomic influences. In contrast, Frame B1 and B2 are from the same time-selected beat-to-beat intervals and illustrate as the short intervals (PP/RR intervals) get shorter (note arrows), the long intervals get longer which is the opposite effect of that shown in frame A. This would be consistent with decremental conduction in the sinoatrial conduction pathways. Additionally, the number of shorter intervals before the longer interval may have an effect (examine beat intervals before longest pause (c). Frame B2 shows the actual ECGs and intervals that are shown in B1 to the duration of the pauses.

Figure SI-4.

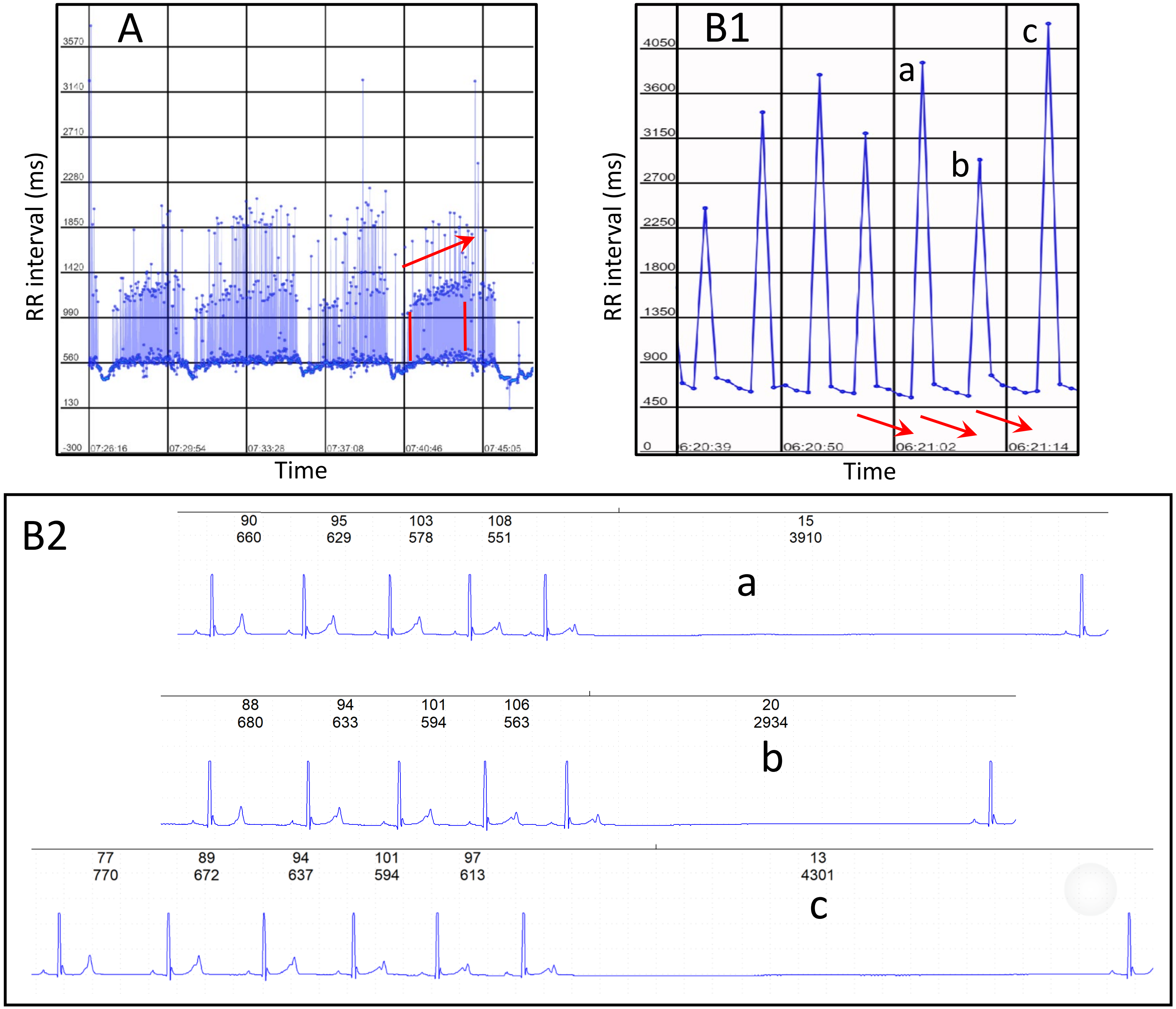
A
RR interval (ms)
3570
3140
2710
2280
1850
1420
990
560
130
-300
07:26:16
07:29:54
07:33:28
07:37:08
07:40:46
07:45:05
Time
B1
RR interval (ms)
4050
3600
3150
2700
2250
1800
1350
900
450
0
6:20:39
06:20:50
06:21:02
06:21:14
a
b
c
Time
B2
90
660
95
629
103
578
108
551
15
3910
a
88
680
94
633
101
594
106
563
20
2934
b
77
770
89
672
94
637
101
594
97
613
13
4301
c

Figure SI-5. Frames A, B and C are from a dynamic three-dimensional Poincaré plot (x = RR interval, y = RR + 1 interval and z = RR + 2) of all training data (~3.5 million intervals). Green represents the balanced autonomic modulation; blue represents high parasympathetic/low sympathetic modulation and red represents sinus node dysfunction. Mixed colors (e.g., purple or rust) represent intervals that overlay with a different classification (intervals with different diagnosis, but same location in three-dimensional space). See SI-Video 4..

Figure SI-5.

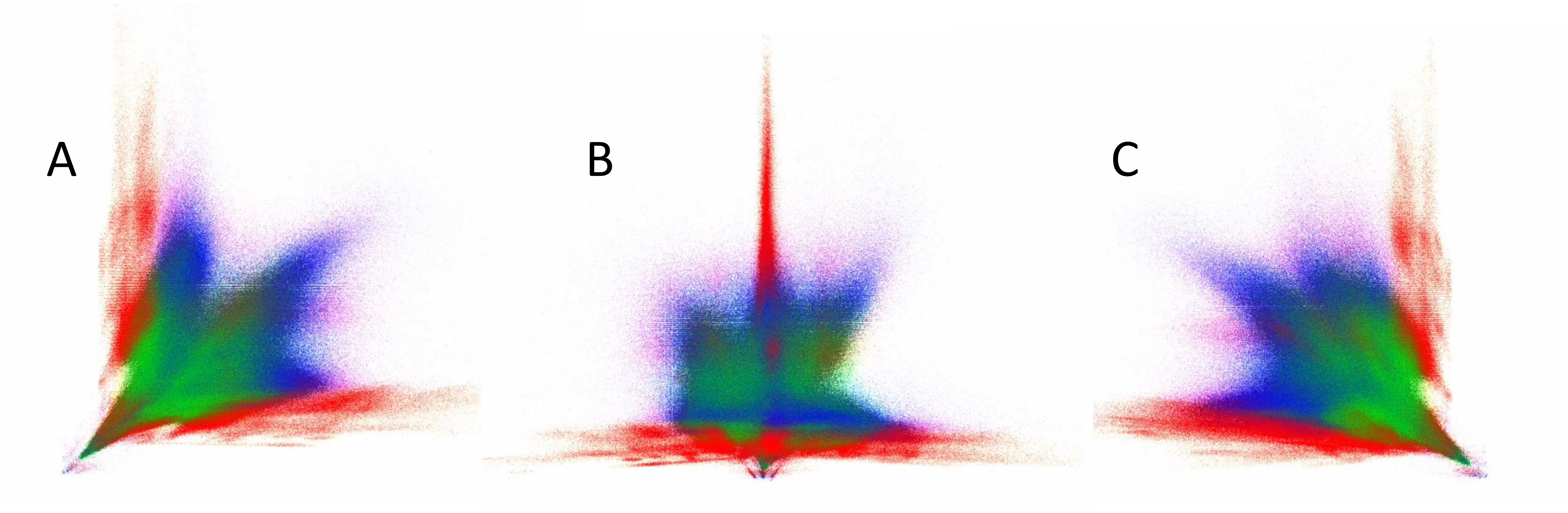

FIGURE SI-6. Example of the classification results of machine learning and the grid classifier of a Holter recording from a dog classified with a balanced autonomic modulation. Both methods made the correct diagnosis ranking balanced autonomic modulation #1. The relative size of the letters indicates the level of indecision for the other two diagnoses. The words and dots have the same color-coding (green, balanced autonomic modulation; blue, high parasympathetic/low sympathetic modulation, and red, sinus node dysfunction). The color-coding of the dots in the tachograms (A) show how each method identified the intervals. Intervals for which the diagnosis was mixed appear as a mixed color (e.g., brown for red and green). Similarly, the Poincaré plots (B) under the tachograms show the distribution by the beat-to-beat interval determination that is then summarized in the 24-hour Poincaré plot shown in (C) with all beat-to-beat intervals overlaid. The three-dimensional plot in the center of frame C shows the beat density of the 24-hour results. Note for clarity the axes for the Poincaré plots is only indicated on this center image. ms – milliseconds.

Figure SI-6.

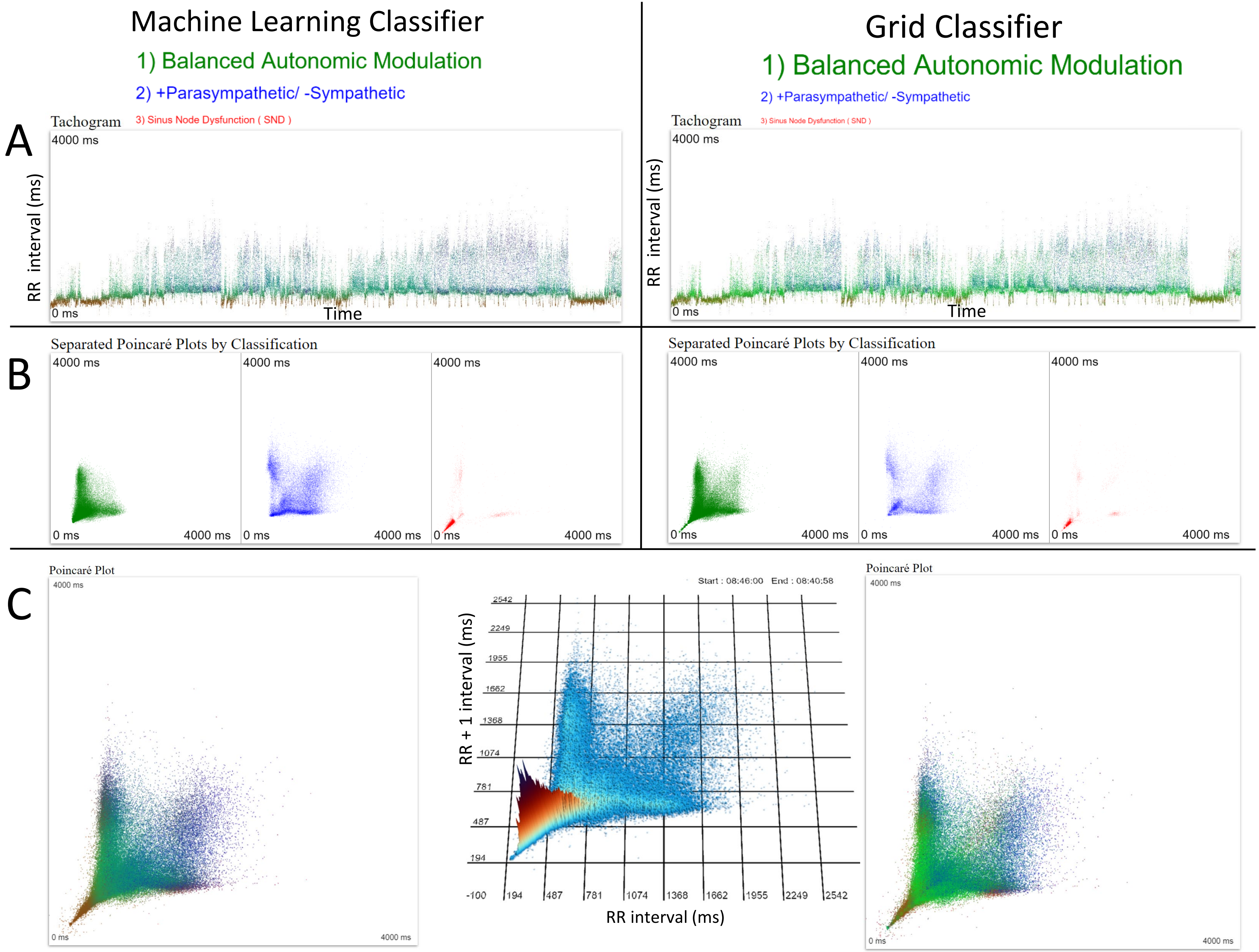
Machine Learning Classifier
1) Balanced Autonomic Modulation
2) +Parasympathetic/ -Sympathetic
3) Sinus Node Dysfunction (SND)
Grid Classifier
1) Balanced Autonomic Modulation
2) +Parasympathetic/ -Sympathetic
3) Sinus Node Dysfunction (SND)
A
Tachogram
4000 ms
RR interval (ms)
0 ms
Time
B
Separated Poincaré Plots by Classification
4000 ms
0 ms
4000 ms
C
Poincaré Plot
Start : 08:46:00 End : 08:40:58
RR + 1 interval (ms)
RR interval (ms)
-100
194
487
781
1074
1368
1662
1955
2249
2542

FIGURE SI-7. Example of the classification results of machine learning and the grid classifier of a Holter recording from a dog classified with a high parasympathetic/low sympathetic modulation. Both methods made the correct diagnosis ranking high parasympathetic/low sympathetic modulation #1. The relative size of the letters indicates the level of indecision for the other two diagnoses. The words and dots have the same color-coding (green, balanced autonomic modulation; blue, high parasympathetic/ low sympathetic modulation, and red, sinus node dysfunction). The color-coding of the dots in the tachograms (A) show how each method identified the intervals. Intervals for which the diagnosis was mixed appear as a mixed color (e.g., purple for red and blue). Similarly, the Poincaré plots (B) under the tachograms show the distribution of the beat-to-beat interval determination that is then summarized in the 24-hour Poincaré plot shown in (C) with all beat-to-beats overlaid. The three-dimensional plot in the center of frame C shows the beat density of the 24-hour results. Note for clarity the axes for the Poincaré plots is only indicated on this center image. ms – milliseconds.

Figure SI-7.

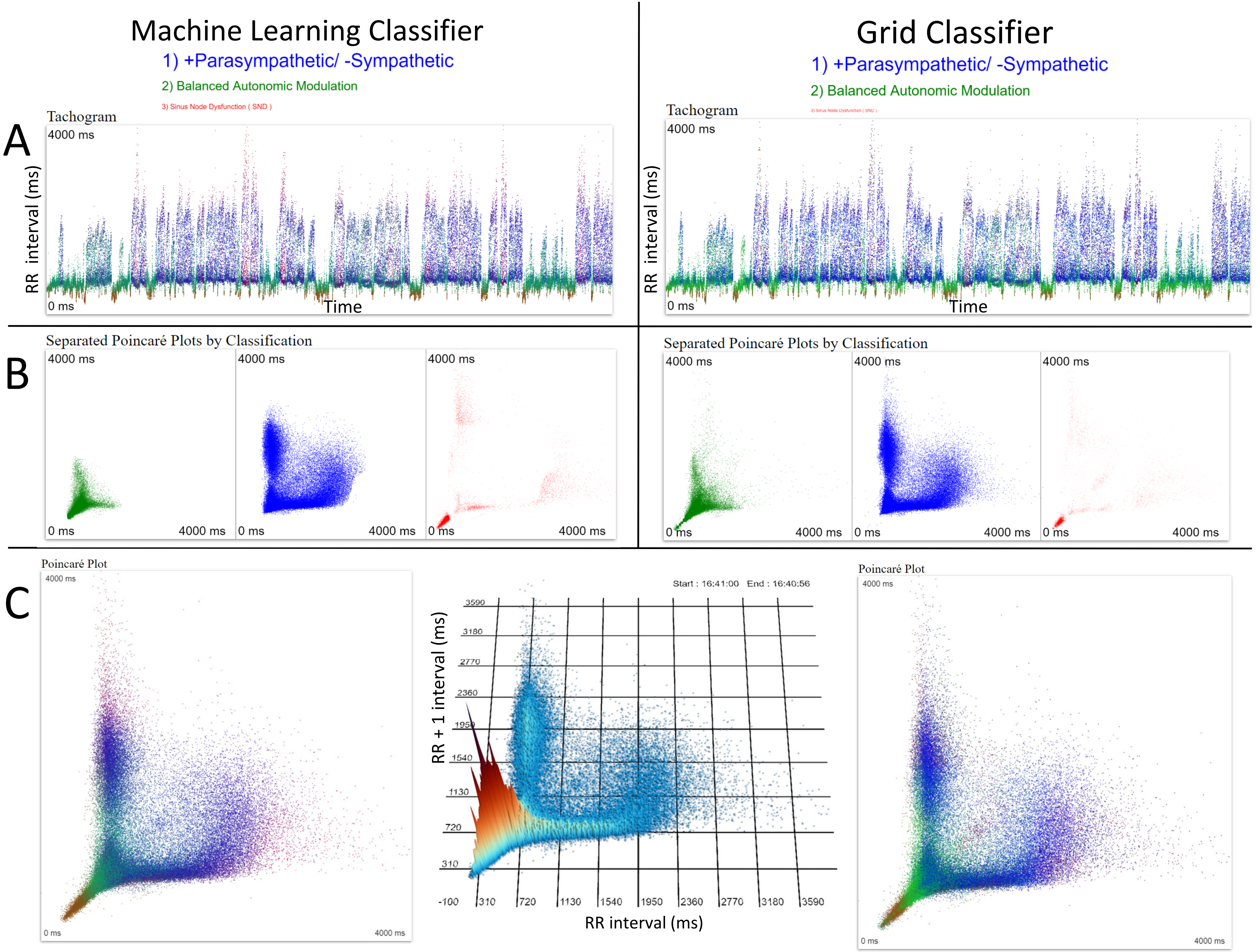
Machine Learning Classifier
1) +Parasympathetic/ -Sympathetic
2) Balanced Autonomic Modulation
3) Sinus Node Dysfunction (SND)
Grid Classifier
1) +Parasympathetic/ -Sympathetic
2) Balanced Autonomic Modulation
A
Tachogram
4000 ms
RR interval (ms)
0 ms
Time
B
Separated Poincaré Plots by Classification
4000 ms
0 ms
C
Poincaré Plot
Start : 16:41:00 End : 16:40:56
RR + 1 interval (ms)
RR interval (ms)
3590
3180
2770
2360
1950
1540
1130
720
310
-100

Figure SI-8. Computer modeling for exit block during conduction from the central sinus node to the atrial myocardium. In the model for high parasympathetic modulation a base rate of 830 ms was used. (A) This interval was derived as the average of those in the lowest band seen on the tachogram. Intervals change by the increasing or decreasing parasympathetic modulation (middle frame) and a randomness factor. As parasympathetic modulation increases, so does the probability of exit block. The exit block probability scale factor, (bottom frame) determines the rate at which block probability increases with parasympathetic tone. The computer-generated intervals during this time (demarcated by the vertical black lines in (A) were then uploaded into the machine learning (B) and grid (C) classifiers. Note the modest differences in the beat-to-beat classification, but the same final diagnosis. Green = balanced autonomic modulation, blue = high parasympathetic modulation, red = sinus node dysfunction. ms = milliseconds.

Figure SI-8.

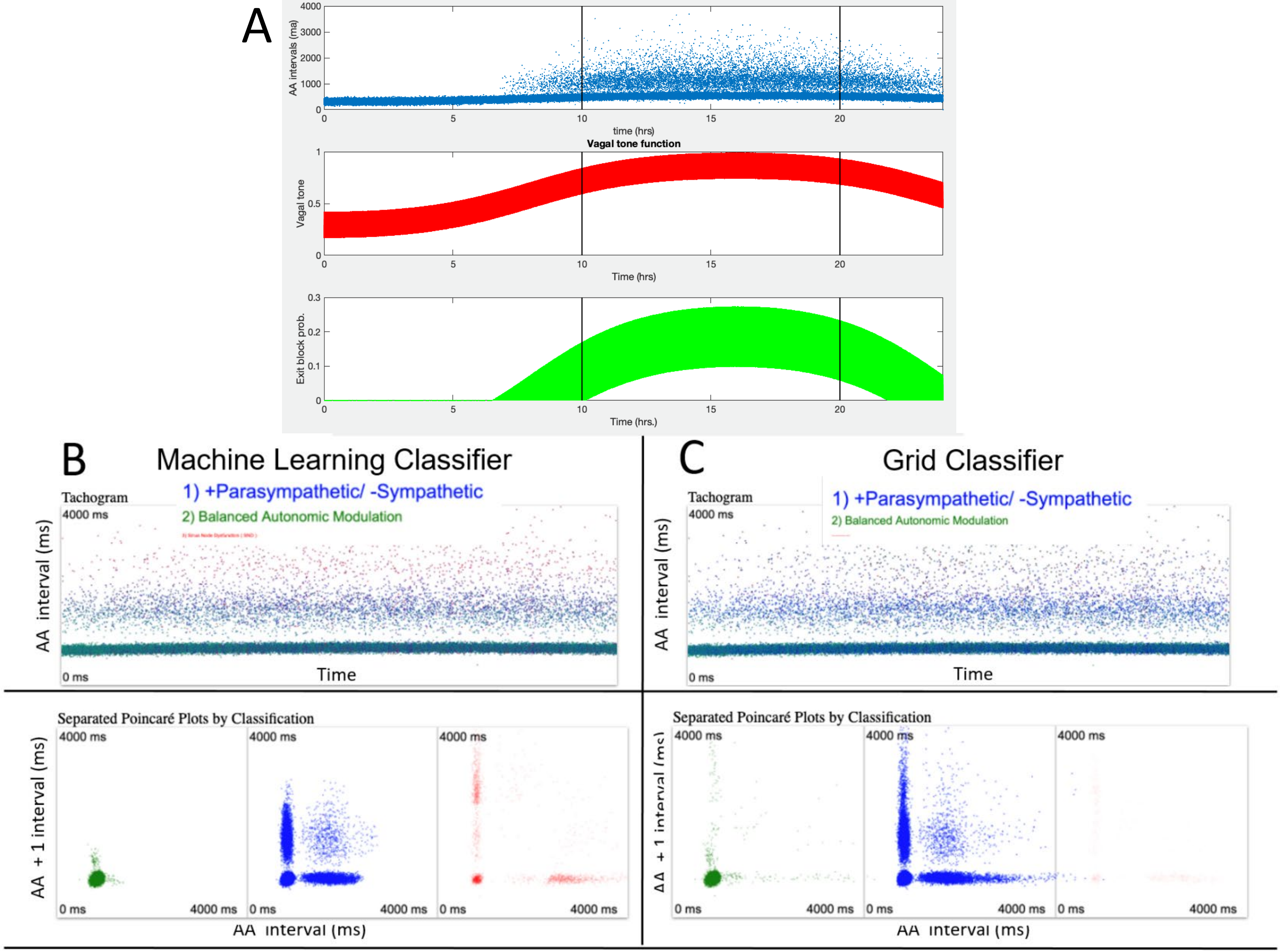

A
AA intervals (ms)
4000
3000
2000
1000
0
0
5
10
15
20
time (hrs)
Vagal tone function
Vagal tone
1
0.5
0
Time (hrs)
Exit block prob.
0.3
0.2
0.1
Time (hrs.)
B
Machine Learning Classifier
Tachogram
1) +Parasympathetic/ -Sympathetic
2) Balanced Autonomic Modulation
4000 ms
AA interval (ms)
0 ms
Time
Separated Poincaré Plots by Classification
AA + 1 interval (ms)
4000 ms
0 ms
AA interval (ms)
C
Grid Classifier
Tachogram
1) +Parasympathetic/ -Sympathetic
2) Balanced Autonomic Modulation
4000 ms
AA interval (ms)
0 ms
Time
Separated Poincaré Plots by Classification
AA + 1 interval (ms)
4000 ms
0 ms
AA interval (ms)
