# Supplementary material for: Use of machine learning and Poincaré density grid in the diagnosis of sinus node dysfunction caused by sinoatrial conduction block in dogs
Source: J Vet Intern Med. 2024 Apr 29;38(3):1305–24. doi: 10.1111/jvim.17071 (PMC11099791; doi:10.1111/jvim.17071)
Supplement: Supplementary file 2 — Table S1. Descriptive data for dogs included in the study. Table S2. Reason given for 24‐hour Holter performed. Table S3. The 24‐hour heart rate data for randomly assigned for training vs testing [median (interquartile values)]. [file JVIM-38-1305-s003.pdf]

SI-Table 1. Descriptive data for dogs included in the study

Variable	Balanced autonomics		High parasympathetic and/or low sympathetic modulation		Sinus node dysfunction	
Weight (kg)	21.7 (9.5-31.4)[a]	n = 23/26[b]	25.5 (22.8-30.2)	n = 19/26	6.2 (4.7-7.6)	n = 7/15
Sex	F/14, M/10[c]	n = 24/26	F/16, M/7	n = 23/26	F/13, M/7	n = 20/21
Age (years)	8 (5-11)[a]	n = 23/26	5.25 (3-7)	n = 24/26	13 (11-14)	n = 19/21
Breeds (number)	Mix n = 5<br>Boxer n = 4<br>Doberman pinscher n = 4<br>Miniature Schnauzer n = 2<br>Unknown n = 2<br>Labrador retriever<br>Rhodesian ridgeback<br>Shih Tzu<br>Boston terrier<br>Italian greyhound<br>Wheaten terrier<br>English Bulldog<br>Beagle<br>Boston terrier		Unknown n = 9<br>German shepherd n = 4<br>Golden retriever n= 2<br>Mix n = 2<br>Boxer n = 2<br>Collie<br>Labradoodle<br>Doberman pincher<br>Labrador retriever<br>Standard poodle<br>Airedale		West Highland White Terrier n = 7<br>Miniature Schnauzer n = 5<br>Mix n = 3<br>Cocker spaniel n = 2<br>Pug n = 2<br>Shih Tzu<br>Bull terrier	

[a] Median (25th-75th quartile)
[b]Data available/total number
[c]F (female)/number, M (male)/number

SI-Table 2 Reason given for 24-hour Holter performed

Reason for Holter	Balanced autonomic modulation n = 26	High parasympathetic and/or low sympathetic modulation n = 26	Sinus node dysfunction n = 21
Syncope or collapse	6	3	9
Question about rhythm based on auscultation	5	2	0
Screening	10	6	2
Bradycardia during anesthesia	1	0	5
Exercise intolerance or weakness	1	0	2
Treatment with beta-adrenergic blocker	0	6	0
Unknown	3	9	3

SI-Table 3 24-hour heart rate data for randomly assigned for training versus testing [median (interquartile values)]

Parameter	Balanced autonomics		High parasympathetic and/or low sympathetic modulation		Sinus node dysfunction		P value
	Training n = 13	Testing n = 13	Training n = 10	Testing n = 16	Training n = 11	Testing n = 10	
Average HR (bpm)	84 (77.5-92.5)	79 (76-89)	61 (57-64)	61 (57.5-62)	67 (59-84)	61.5 (51.5-68.5)	NS
Average RR interval (ms)	714 (649-774)	759 (675-789)	984 (938-1053)	984 (968-1044)	895 (714-1017)	976 (876-1165)	NS
Minimum HR (bpm)	45 (42-52)	43 (41-46)	32.5 (30.8-35.3)	32.5 (29.5-34)	28 (24-30)	25.5 (22-26.5)	NS
Time <50 bpm (min)	1.9 (0-26.1)	13.8 (1.0-55.7)	567.5 (346.5-798)	606 (516-700.5)	318 (132-674)	532 (270-783)	NS
Number of pauses >2 s	41 (6-80)	45 (27-215)	3281 (477-5738)	1921 (743-3716)	7972 (6317-12,000)	9010 (7091-9998)	NS
Number of pauses >3 s	0 (0-1.5)	0 (0-2.5)	23 (0-156.3)	67 (1.5-109.8)	1668 (412-4463)	2948 (1211-4255)	NS
Number of pauses >4 s	0 (0-0)	0 (0-0)	0 (0-4.5)	0 (0.3-14.5)	158 (48-198)	330 (68-575)	NS
Longest pause (s)	2.7 (2.2-3.4)	2.8 (2.6-3.3)	3.5 (2.8-4.4)	4.3 (3.2-5)	6.3 (5.5-7.9)	8.4 (5.5-12.2)	NS
cRMSSD (ms)	0.459 (0.418-0.506)	0.426 (0.397-0.467)	0.507 (0.448-0.621)	0.470 (0.413-0.506)	0.787 (0.733-0.938)	0.780 (0.712-0.932)	NS

HR, heart rate; bpm, beats per minute; s, seconds; ms, milliseconds; min, minutes; cRMSSD, heart rate corrected root mean square of successive RR intervals; NS, not significantly different
