# Supplementary material for: Use of machine learning and Poincaré density grid in the diagnosis of sinus node dysfunction caused by sinoatrial conduction block in dogs
Source: J Vet Intern Med. 2024 Apr 29;38(3):1305–24. doi: 10.1111/jvim.17071 (PMC11099791; doi:10.1111/jvim.17071)
Supplement: Supplementary file 3 — Video S1. Comparison of dynamic Poincaré plots from a dog with sinus node dysfunction (red) and a dog with high parasympathetic modulation (blue). Note beat‐to‐beat pattern differences during selected‐times when dogs had similar heart rates (300 beat‐to‐beat intervals shown at a time with a change of 100 beats/second). This video compliments Figure 4. Video S2. Two different selected times during the 24‐hour recordings from 2 dogs overlaid for comparison. Dog in red has sinus node dysfunction‐1 and dog in gold has sinus node dysfunction‐2. (300 beat‐to‐beat intervals shown at a time with a change of 100 beats/second). This video compliments Figure 10. Video S3. Video of 3‐dimensional dynamic map created from the neural network (machine learning) trained dataset. This plot gives all possible outputs of the neural network. Green represents the balanced autonomic modulation; blue represents HP/LSM and red represents sinus node dysfunction. RR = beat‐to‐beat interval. This video corresponds to Figure 12. Video S4. Dynamic 3‐dimensional Poincaré plot of all training data (~3.5 million intervals). Green represents the balanced autonomic modulation, blue represents HP/LSM and red represents sinus node dysfunction. Mixed colors (eg, purple or rust) represent intervals that overlay with a different classification (intervals with different diagnosis, but same location in 3‐dimensional space). RR = beat‐to‐beat interval. This video corresponds to Figure 13. [file JVIM-38-1305-s002.pptx]

## Slide 1
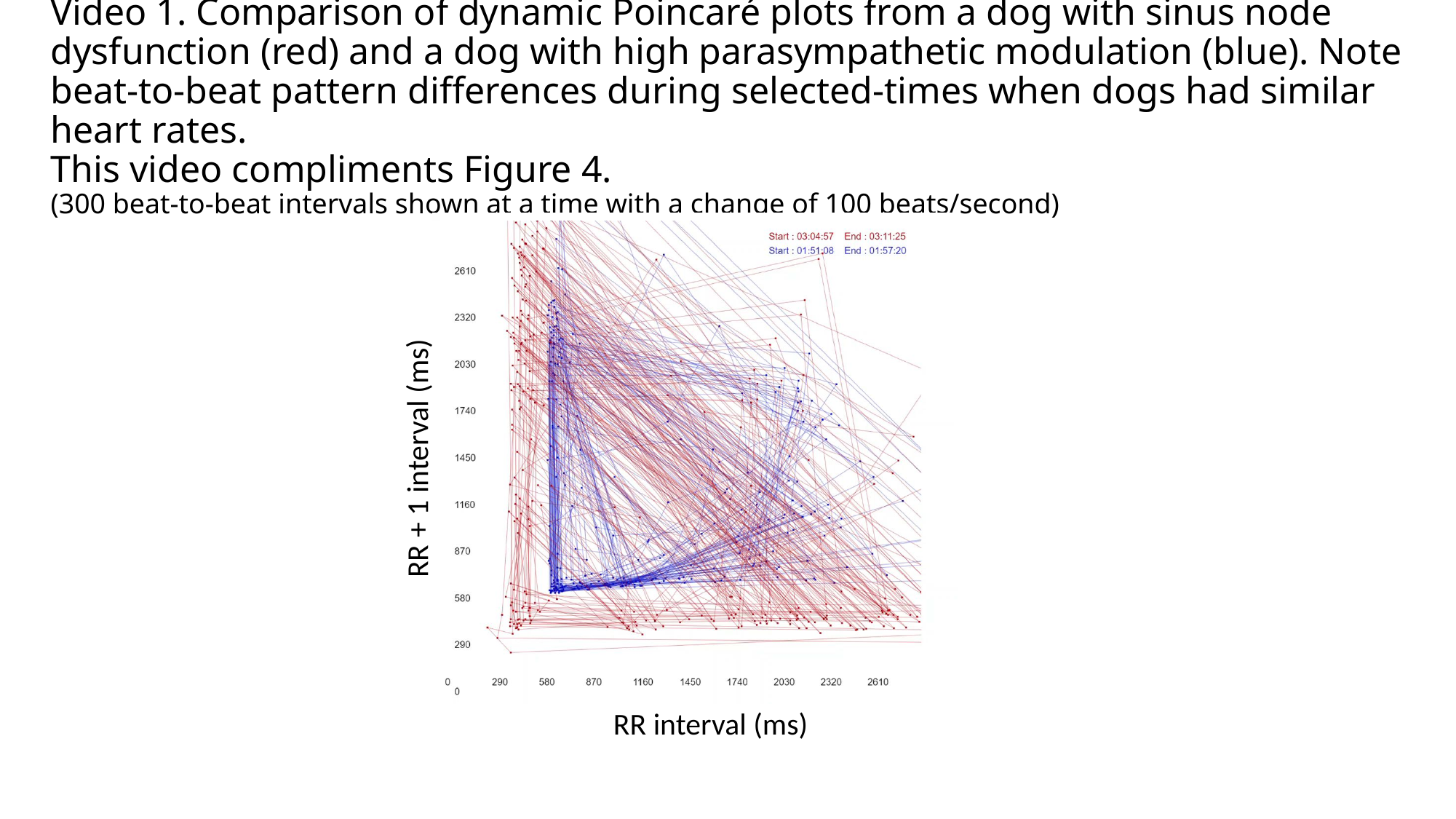

# Video 1. Comparison of dynamic Poincaré plots from a dog with sinus node dysfunction (red) and a dog with high parasympathetic modulation (blue). Note beat-to-beat pattern differences during selected-times when dogs had similar heart rates. This video compliments Figure 4. (300 beat-to-beat intervals shown at a time with a change of 100 beats/second)
RR + 1 interval (ms)
RR interval (ms)

## Slide 2
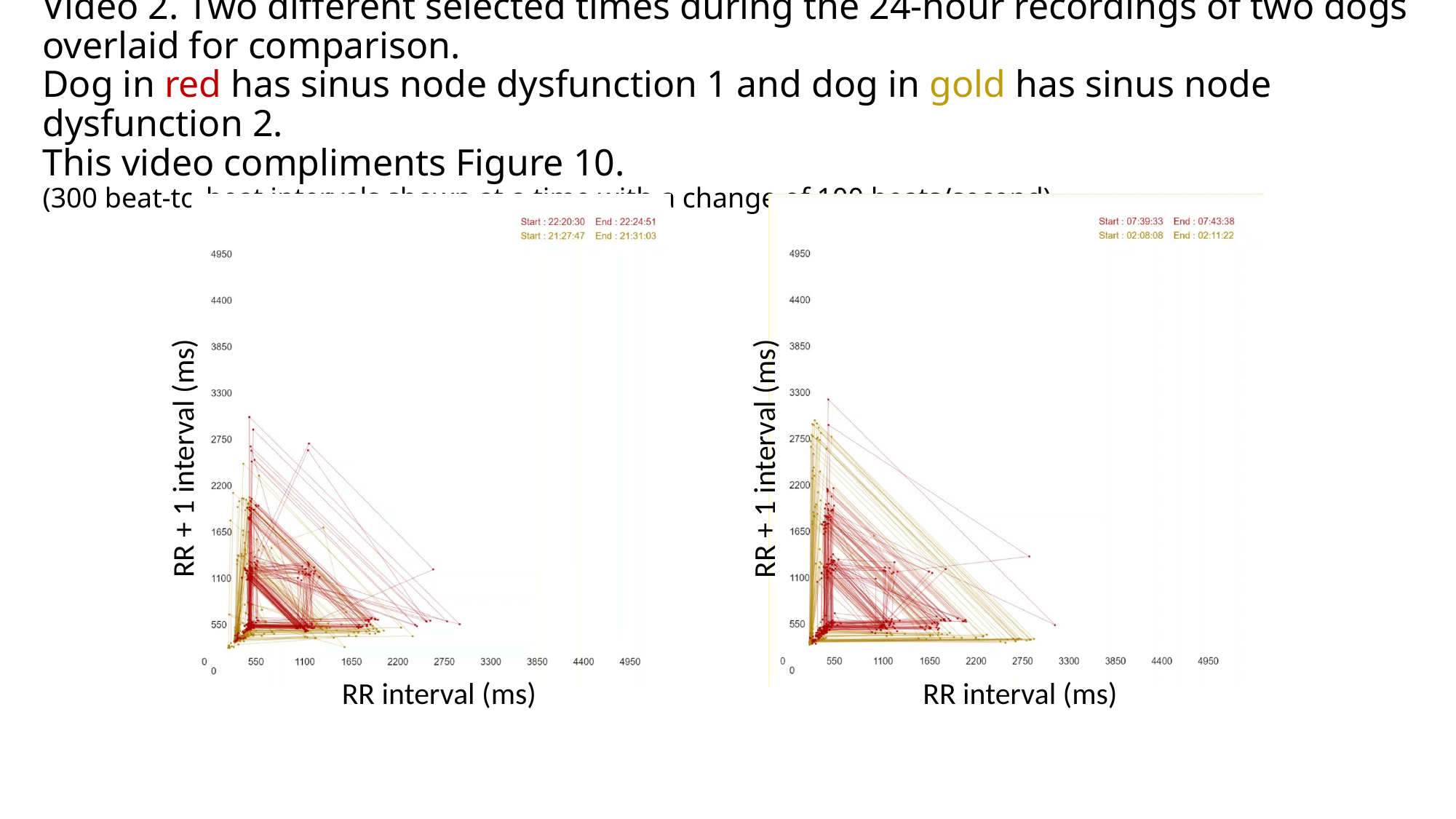

# Video 2. Two different selected times during the 24-hour recordings of two dogs overlaid for comparison. Dog in red has sinus node dysfunction 1 and dog in gold has sinus node dysfunction 2. This video compliments Figure 10. (300 beat-to-beat intervals shown at a time with a change of 100 beats/second)
RR + 1 interval (ms)
RR + 1 interval (ms)
RR interval (ms)
RR interval (ms)

## Slide 3
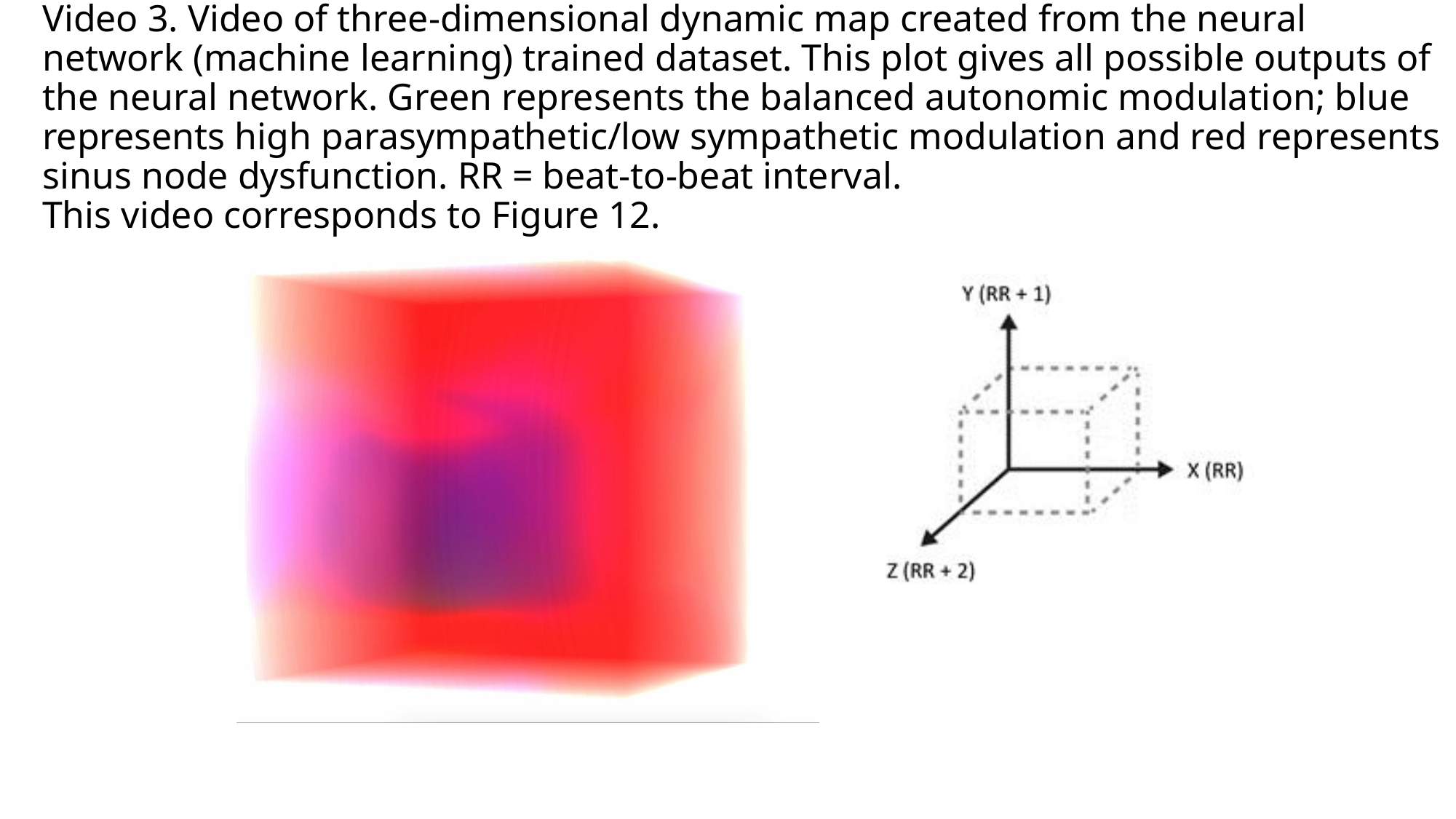

# Video 3. Video of three-dimensional dynamic map created from the neural network (machine learning) trained dataset. This plot gives all possible outputs of the neural network. Green represents the balanced autonomic modulation; blue represents high parasympathetic/low sympathetic modulation and red represents sinus node dysfunction. RR = beat-to-beat interval. This video corresponds to Figure 12.

## Slide 4
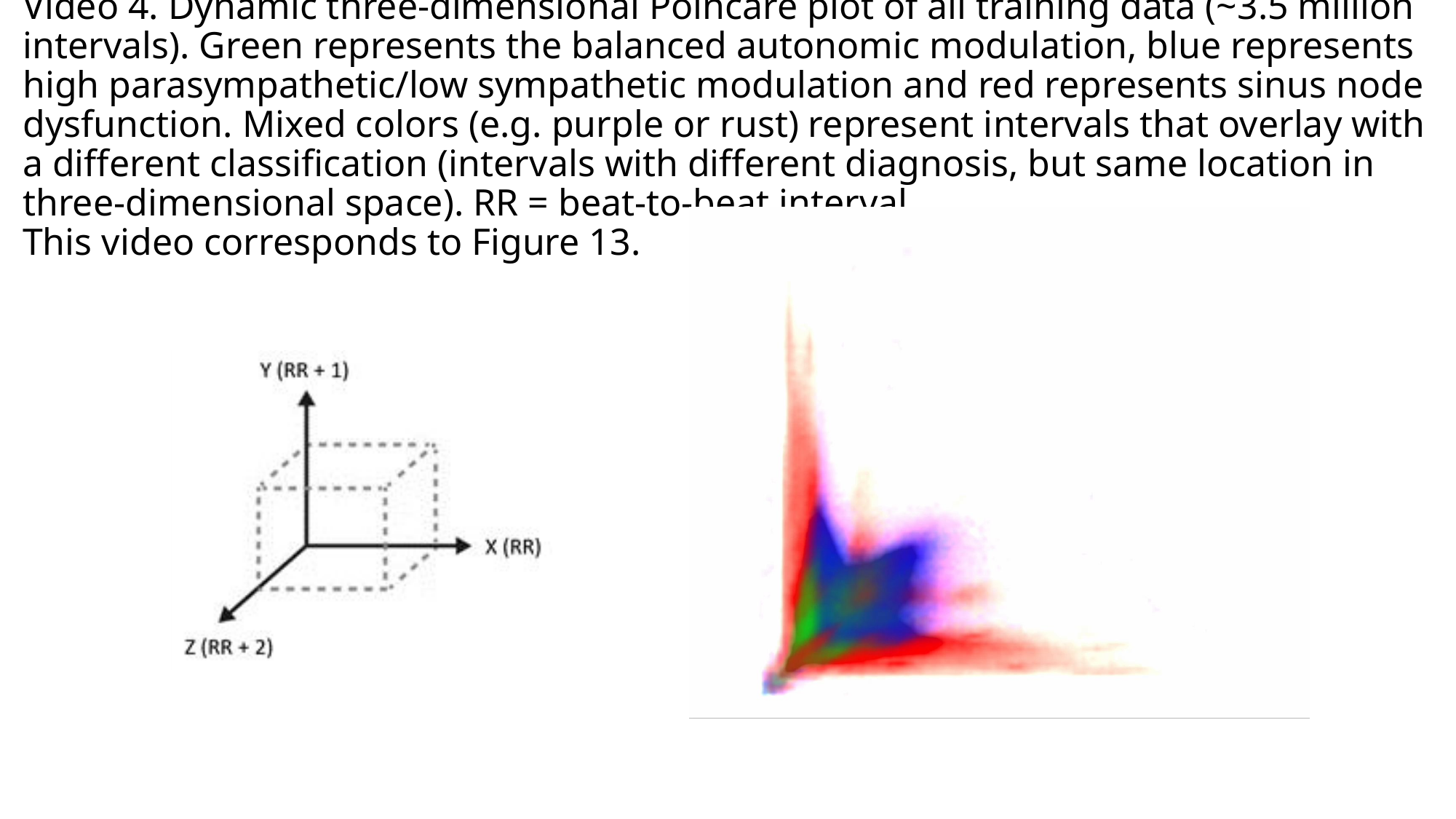

# Video 4. Dynamic three-dimensional Poincaré plot of all training data (~3.5 million intervals). Green represents the balanced autonomic modulation, blue represents high parasympathetic/low sympathetic modulation and red represents sinus node dysfunction. Mixed colors (e.g. purple or rust) represent intervals that overlay with a different classification (intervals with different diagnosis, but same location in three-dimensional space). RR = beat-to-beat interval. This video corresponds to Figure 13.
